# Autism-Risk Gene *necab2* Regulates Psychomotor and Social Behavior as a Neuronal Modulator of mGluR1 Signaling

**DOI:** 10.3389/fnmol.2022.901682

**Published:** 2022-07-13

**Authors:** Zexu Chen, Han Long, Jianhua Guo, Yiran Wang, Kezhe He, Chenchen Tao, Xiong Li, Keji Jiang, Su Guo, Yan Pi

**Affiliations:** ^1^State Key Laboratory of Genetic Engineering, School of Life Sciences, Fudan University, Shanghai, China; ^2^National Demonstration Center for Experimental Biology Education, School of Life Sciences, Fudan University, Shanghai, China; ^3^Eye Institute and Department of Ophthalmology, Eye & ENT Hospital, Fudan University, Shanghai, China; ^4^NHC Key Laboratory of Myopia (Fudan University), Key Laboratory of Myopia, Chinese Academy of Medical Sciences, Shanghai, China; ^5^School of Clinical Medicine, Shanghai Medical College, Fudan University, Shanghai, China; ^6^Department of Bioengineering and Therapeutic Sciences, Programs in Human Genetics and Biological Sciences, University of California, San Francisco, San Francisco, CA, United States; ^7^Department of Pulmonary and Critical Care Medicine, Zhongshan Hospital, Fudan University, Shanghai, China; ^8^East China Sea Fisheries Research Institute, Chinese Academy of Fishery Sciences, Shanghai, China

**Keywords:** autism spectrum disorders, calcium-binding protein, social behavior, metabotropic glutamate receptor 1, cerebellum

## Abstract

**Background:**

*De novo* deletion of the *neuronal calcium-binding protein 2* (*NECAB2*) locus is associated with idiopathic autism spectrum disorders (ASDs). The *in vivo* function of *NECAB2* in the brain remains largely elusive.

**Methods:**

We investigated the morphological and behavioral profiles of both *necab2* knock-out and overexpression zebrafish models. The expression pattern and molecular role of *necab2* were probed through a combination of *in vitro* and *in vivo* assays.

**Results:**

We show that Necab2 is a neuronal specific, cytoplasmic, and membrane-associated protein, abundantly expressed in the telencephalon, habenula, and cerebellum. Necab2 is distributed peri-synaptically in subsets of glutamatergic and GABAergic neurons. CRISPR/Cas9-generated *necab2* knock-out zebrafish display normal morphology but exhibit a decrease in locomotor activity and thigmotaxis with impaired social interaction only in males. Conversely, *necab2* overexpression yields behavioral phenotypes opposite to the loss-of-function. Proteomic profiling uncovers a role of Necab2 in modulating signal transduction of G-protein coupled receptors. Specifically, co-immunoprecipitation, immunofluorescence, and confocal live-cell imaging suggest a complex containing NECAB2 and the metabotropic glutamate receptor 1 (mGluR1). *In vivo* measurement of phosphatidylinositol 4,5-bisphosphate further substantiates that Necab2 promotes mGluR1 signaling.

**Conclusions:**

Necab2 regulates psychomotor and social behavior *via* modulating a signaling cascade downstream of mGluR1.

## Introduction

Human genetics have uncovered many genes associated with complex neurodevelopmental disorders including intellectual disability, autism spectrum disorders (ASD), and schizophrenia (Sanders et al., 2011; De Rubeis et al., 2014; Iossifov et al., 2014; Schizophrenia Working Group of the Psychiatric Genomics Consortium, 2014; Niemi et al., 2018). The major challenges ahead are to establish efficient and cost-effective approaches to evaluate the mechanisms by which alterations of these genes may cause brain abnormalities, and devise potential therapeutic strategies harnessing this knowledge.

Zebrafish is a promising model system to perform such studies. As a vertebrate, zebrafish share considerable genomic similarities with mammals. There are zebrafish orthologues for about 82% of genes associated with human diseases (Howe et al., 2013). Also, tools for loss- (Hsu et al., 2014) and gain-of-function studies (Kawakami et al., 2016) have been well-established. Chemical treatment and behavioral tracking can be conveniently implemented in high-throughput studies, thereby linking genes to the brain and behavior (Guo, 2004; Thyme et al., 2019). Neural activity can be measured brain-wide at single-cell resolution (Chen et al., 2018).

Calcium is an important regulator of neuronal activity. Its dysregulation has been linked to neurological diseases (Schwaller, 2020). Calcium influx triggers exocytosis of neurotransmitter-containing synaptic vesicles at the presynaptic terminals (Neher and Sakaba, 2008). A transient rise of calcium levels in the dendritic spines post-synaptically induces activity-dependent synaptic plasticity (Zucker, 1999). Calcium signals can also regulate gene transcription at a slower time scale ranging from minutes to hours (Lyons and West, 2011). Complementing the omnipotence of Ca^2+^ as a neuronal regulator are a variety of calcium-binding proteins. *Neuronal Ca^2+^ binding proteins* (*NECABs*) are a protein family characterized by a single N-terminal EF-hand domain responsible for calcium binding, a putative antibiotic biosynthesis monooxygenase (ABM) domain at the C terminus, and a NECAB homology region (NHR) in between (Sugita et al., 2002; Kawasaki and Kretsinger, 2017). At least three members (*NECAB1-3*) are identified in humans that are conserved across vertebrates. NECABs are either expressed primarily in the nervous system (*NECAB1* and *NECAB2*) or the brain and muscle (*NECAB3*) (Zimmermann et al., 2013). *NECAB2* has been reported to interact with two G-protein coupled receptors in the human embryonic kidney cells in a calcium-regulated manner (Canela et al., 2007, 2009). At the spinal level, *necab2* is down-regulated by the peripheral nerve injury (Zhang et al., 2014) and functions as a critical determinant of pro-nociceptive neurotransmission (Zhang et al., 2018; Ma et al., 2021). A proteomic study involving yeast two-hybrid screening showed that *NECAB2* interacts with five autism-risk genes (*FMR1*, *FXR1*, *FXR2*, *SMARCA2*, and *TSC1*) (Sakai et al., 2011). A *de novo* deletion spanning the locus of *NECAB2* has been clinically detected in patients with idiopathic ASD (Itsara et al., 2010; Sakai et al., 2011; Sanders et al., 2011). However, the *in vivo* function of *NECAB2* in the brain remains elusive. As such, it remains speculative whether and how *NECAB2* might be involved in the pathogenesis of ASD.

In the present study, we performed *in vivo* functional analysis of *necab2* employing zebrafish. By generating knock-out and isoform-specific overexpression models, we showed that *necab2* played a critical role in regulating psychomotor and social behaviors. We further uncovered the molecular role that Necab2 interact with metabotropic glutamate receptor subtype 1 (mGluR1) and promote phosphatidylinositol 4,5-bisphosphate hydrolysis. These findings lay foundation for devising potential therapeutic strategies to combat the neurodevelopmental disorders involving *NECAB2* and its interacting molecular pathways.

## Results

### *necab2* Is Conserved in the Vertebrate Lineage and Expressed in the Developing Brain

**FIGURE 1:**
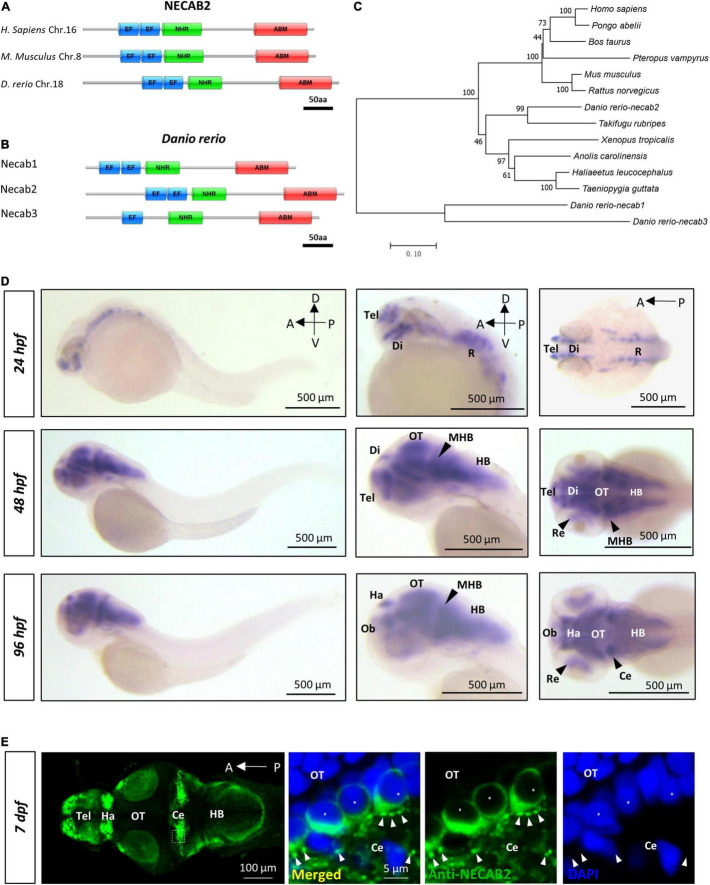
*necab2*, conserved in the vertebrate lineage, is expressed in the developing zebrafish brain. **(A)** Protein Diagrams of NECAB2 orthologs in *H. sapiens*, *M. musculus*, and *D. rerio*. Diagrams represented the longest isoform. The diagrams were denoted by the species name and chromosome number (Chr.) All major functional domains are conserved. Scale bar = 50 amino acids (aa). **(B)** Protein Diagrams of the Necab family in *D. rerio*. Diagrams represented the longest isoforms. Diagrams are denoted by the gene name. All the major functional domains were conserved in Necab1, Necab2, and Necab3. Scale bar = 50 amino acids (aa). **(C)** The phylogenetic tree of the evolutionary relationship of *NECAB2*. Diverse animal phyla were identified by a best reciprocal BLAST search with human *NECAB2* and were mapped onto a phylogenetic tree. The tree was drawn to scale, with branch lengths in the same units as those of the evolutionary distances. All of the sequences were available from the NCBI protein database. Scale bar = 0.10. **(D)** Whole-mount *in situ* hybridization of *necab2* in wild-type embryos at 24, 48, and 96 hpf. *necab2* was expressed in selected brain regions reminiscent of the locations of nascent neurons. Scale bar = 500 μm. **(E)** Whole-mount immunofluorescence of Necab2 in wild-type larvae at 7 dpf. Necab2 was abundant in the telencephalon, habenula, and cerebellum. Scale bar = 100 μm. Necab2 was detected in structures reminiscent of synapse boutons (arrowheads) and the cell cytoplasm/membrane avoiding the nucleus (asterisks). The region in the dashed white box was shown at a higher magnification on the right. Scale bar = 5 μm. NECAB2 protein domains: EF, EF-Hand; NHR, NECAB Homologous Region; ABM, Antibiotic Biosynthesis Monooxygenase. hpf, hour post-fertilization; dpf, day post-fertilization; Tel, telencephalon; Di, diencephalon; Ce, cerebellum; Re, retina; Ha, habenula; OT, optic tectum; MHB, midbrain-hindbrain boundary; HB, hindbrain.

NECAB2 belongs to a protein family with three recognizable domains: the EF-hand at the N-terminus, the middle NHR domain, and an ABM motif at the C-terminus (Figure 1A). In zebrafish, all three functional domains were conserved among Necab1-3, all of which have only one ortholog in the zebrafish genome (Figure 1B). The Necab2 protein sequence shares 44 and 38% of amino acid identity with Necab1 and Necab3. Multiple transcripts of *necab2* have been predicted in the Ensemble database (Supplementary Figure 1A). Two isoforms of Necab2 were confirmed at both RNA and protein levels. One of them possesses alternative splicing in the exon 9, which generated a premature stop codon before translating the ABM motif, named *necab2-201* (Supplementary Figures 1B,C). A phylogenetic analysis of the *necab2* orthologs across species revealed that NECAB2 is presented in *Homo sapiens* and at least 12 other vertebrates (Figure 1C) but is undetectable in the invertebrates such as *Drosophila* or *Caenorhabditis elegans*. The Necab2 protein sequences of zebrafish exhibit a relatively high level of amino acid identity with the corresponding human protein and the three typical conserved domains (EF-hand, NHR, ABM) exhibit 81, 86, and 60% amino acid identity respectively. Together, these findings indicate that NECAB2 is conserved in vertebrates and zebrafish is a potential model for studying the *in vivo* function of Necab2 in the intact brain.

The distribution of NECAB2 has been well-characterized in the spinal cord and peripheral nervous system (Zhang et al., 2014, 2016). However, its distribution in the brain remains largely unexplored. Using whole-mount *in situ* hybridization in embryonic and larval zebrafish, we found that *necab2* was expressed in the retina and subsets of brain regions examined from 24 hpf (hours post fertilization) to 96 hpf, resembling the distribution of nascent neurons (Figure 1D). Immunofluorescent labeling studies showed that the distribution of Necab2 protein was similar to its RNA transcripts but had stronger enrichment in the telencephalon, habenula, and cerebellum (Figure 1E). Subcellularly, immunofluorescent staining indicated Necab2 to be enriched in structures resembling the synapses (Figure 1E). All the RNA probes and antibodies used in this study were verified (Supplementary Figure 2). Thus, in contrast to other calcium-binding proteins that confine to cytoplasmic domains, these results suggest that Necab2 might not be merely a cytoplasmic calcium buffer.

### *necab2* Is Expressed Peri-Synaptically in Subsets of Glutamatergic and GABAergic Neurons

**FIGURE 2:**
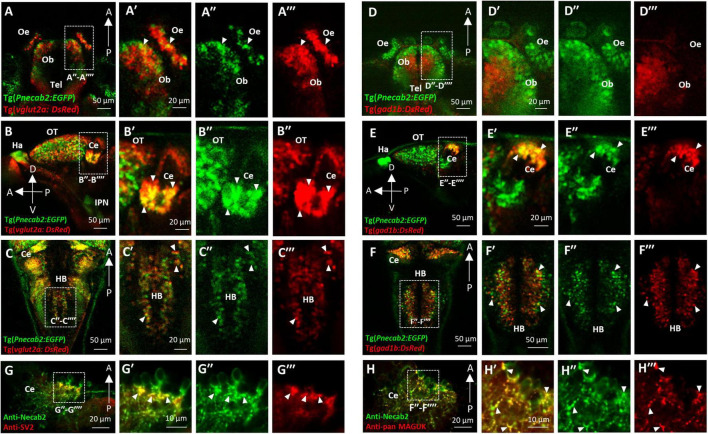
Necab2 is expressed in subsets of glutamatergic and GABAergic neurons. **(A–A″′)** Confocal live imaging of the transgenic fish Tg(*Pnecab2:EGFP*) crossed with Tg(*vglut2a:DsRed*) at 5 dpf. Necab2 was expressed in subsets of glutamatergic neurons in the telencephalon, olfactory bulb, and olfactory epithelium (arrowheads, dorsal views). Scale bar = 50 μm **(A)**. The region in the dashed white box **(A)** was shown at higher magnification on the right **(A′–A″′)**. Scale bar = 20 μm. **(B–B″′)**. Necab2 was expressed in subsets of glutamatergic neurons in the optic tectum and cerebellum (arrowheads, lateral views). Scale bar = 50 μm **(B)**. The region in the dashed white box **(B)** was shown at higher magnification on the right **(B′–B″′)**. Scale bar = 20 μm. **(C–C″′)** Necab2 was expressed in subsets of glutamatergic neurons in the hindbrain (arrowheads, dorsal views). Scale bar = 50 μm **(C′)**. The region in the dashed white box **(C)** was shown at higher magnification on the right **(C′–C″′)**. Scale bar = 20 μm. **(D–D″′)** Confocal live imaging of the transgenic fish Tg(*Pnecab2:EGFP*) crossed with Tg(*gad1b:DsRed*) at 5 dpf. The Necab2-expressing neurons rarely overlapped with the gad1b-positive neurons in the telencephalon, olfactory bulb, or olfactory epithelium (arrowheads, dorsal views). Scale bar = 50 μm **(D)**. The region in the dashed white box **(D)** was shown at higher magnification on the right **(D′–D″′)**. Scale bar = 20 μm. **(E–E″′)** Necab2-expressing neurons strongly overlapped with gad1b-positive neurons in the cerebellum (arrowheads, lateral views). Scale bar = 50 μm **(E)**. The region in the dashed white box **(E)** was shown at higher magnification on the right **(E′–E″″)**. Scale bar = 20 μm. **(F–F″′)** Necab2-expressing neurons overlapped with gad1b-positive neurons in the hindbrain (arrowheads, dorsal views). Scale bar = 50 μm **(F)**. The region in the dashed white box **(F)** was shown at higher magnification on the right **(F′–F″′)**. Scale bar = 50 μm. **(G–G″′)** Co-immunofluorescent staining of the anti-Necab2 with anti-SV2 in 7 dpf larvae showed that Necab2 was enriched in the pre-synaptic structures (arrowheads). Scale bar = 20 μm **(G)**. The region in the dashed white box **(G)** is shown at higher magnification on the right **(G′–G″′)**. Scale bar = 10 μm. **(H–H″′)** Co-immunofluorescent staining of anti-Necab2 with anti-pan MAGUK in the 7 dpf larvae showed that Necab2 was enriched in the postsynaptic structures (arrowheads). Scale bar = 20 μm **(H)**. The region in the dashed white box **(H)** was shown at higher magnification on the right **(H′–H″′)**. Scale bar = 10 μm. dpf, day post-fertilization; Tel, telencephalon; Ce, cerebellum; Hb, habenula; OT, optic tectum; IPN, interpeduncular nucleus.

To further characterize Necab2 expression at the cellular level, we established a transgenic reporter line, Tg (*Pnecab2:EGFP*), expressing EGFP under the control of upstream regulatory elements of the *necab2* gene. The fidelity of transgene expression was verified (Supplementary Figures 3A,B), and the EGFP signal was detectable at single-cell resolution (Supplementary Figure 3C). By crossing the reporter line with those that labeled glutamatergic [Tg(*vglut2a:DsRed*)], GABAergic [Tg(*gad1b:DsRed*)], and glycinergic [Tg(*glyt2:DsRed*)] neurons as well as performing immunofluorescent labeling of dopaminergic (anti-TH) and serotonergic (anti-5-HT) neurons, we found that *necab2* was expressed in subsets of glutamatergic and GABAergic (Figure 2) but not in other types of neurons (Supplementary Figure 4). In the glutamatergic neurons, Necab2 co-localized with Vglut2a in sub-regions of the telencephalon (Figure 2A), diencephalon, tectum, cerebellum (Figure 2B), and hindbrain (Figure 2C). Notably, the overlap between *necab2*-expressing and *gad1b*-expressing neurons was not detectable in the forebrain (Figure 2D) but was prominent in the cerebellum (Figure 2E), and the hindbrain (Figure 2F). We also examined whether Necab2 was located pre-synaptically (labeled by anti-SV2) or post-synaptically (labeled by anti-pan MAGUK). Necab2 was enriched in both the pre-synaptic (Figure 2G) and post-synaptic structures (Figure 2H) in the cerebellum. Taken together, Necab2 displays restricted expression in subsets of excitatory and inhibitory neuronal terminals, suggesting that it might play a role in modulating synaptic signaling.

### Generation of a *necab2* Mutant *via* CRISPR/Cas9 and *necab2* Over-Expressing Lines *via in vivo* Transgenesis

**FIGURE 3:**
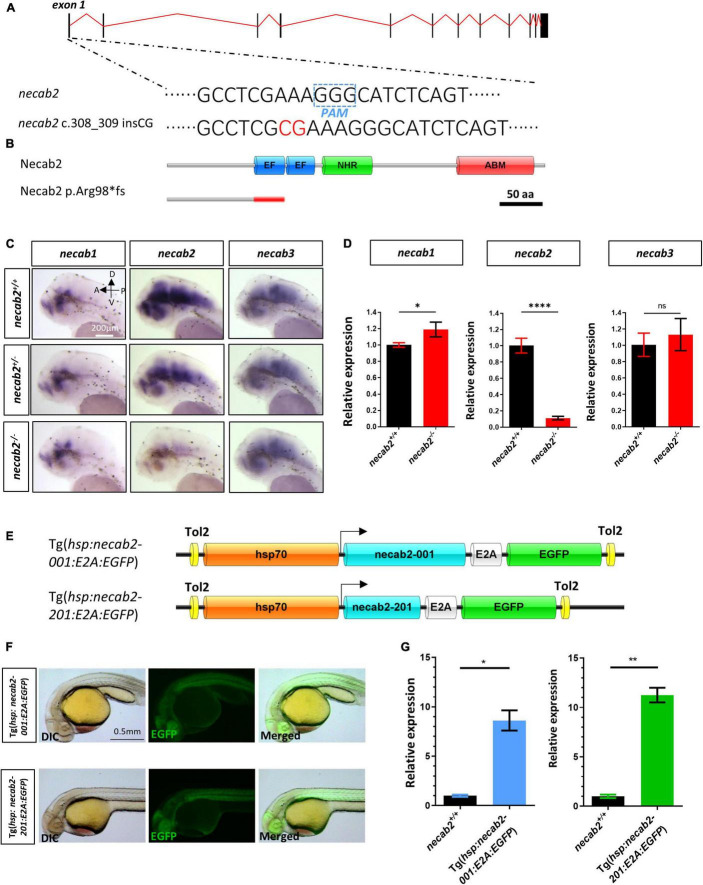
Generation of a *necab2* mutant *via* CRISPR/Cas9 and necab2 over-expression lines by Tol2 transgenesis. **(A)** Schematic presentation of the genomic structure of *necab2* and a 2 bp insertion in exon1 generated by gene targeting *via* CRISPR. The PAM site was highlighted in the dashed blue box. The inserted base was shown in red. **(B)** Predicted structures of Necab2^+/+^ and Necab2*^–^*^/^*^–^* proteins in zebrafish. The 2 bp insertion in exon 1 resulted in a frame-shift mutation and premature termination before the EF-hand domain. **(C)** Spatial analysis of *necab1*, *necab2*, and *necab3* mRNA by whole-mount *in situ* hybridization was performed blindly in the embryos derived from the in-cross of *necab2*^+/–^ followed by genotyping. Scale bar = 200 μm. **(D)** Quantitative analysis of *necab1*, *necab2*, and *necab3* mRNA by qRT-PCR. The mRNA of *necab3* did not show a significant compensation effect while the upregulation of the *necab1* mRNA reached statistical significance. (ns *p* ≥ 0.05, *p < 0.05, ****p < 0.0001, Student’s *t*-test or Mann-Whitney *U* test). **(E)** Schematic presentation of the *Tol2* transgenic construction of Tg(*hsp:necab2-001:E2A:EGFP*) and Tg(*hsp:necab2-201:E2A:EGFP*). **(F)** Fluorescent imaging of the transgenic fish Tg(*hsp:necab2-001:E2A:EGFP*) and Tg(*hsp:necab2-201:E2A:EGFP*). Fish were processed for 37°C heat-shock for 1 h at 1 dpf. Scale bar = 0.5 mm. **(G)** Quantitative analysis of the *necab2* mRNA in Tg(*hsp:necab2-001:E2A:EGFP*) and Tg(*hsp:necab2-201:E2A:EGFP*) against their wild-type counterparts respectively by qRT-PCR. (*p < 0.05, **p < 0.01, Student’s *t* test or Mann–Whitney U test.) dpf, day post-fertilization.

The function of *necab2* was further elucidated by knocking out the gene using CRISPR/Cas9 genome editing. A mutant allele was generated, containing 2 bp insertion (c.308_309 insCG) in front of the EF-hand domain (Figure 3A), resulting in a frameshift and a putative premature stop codon (p.Arg98*fs) (Figure 3B). Applying *in situ* hybridization and qPCR, we detected a significant decrease in *necab2* transcript levels in the mutant larvae (Figures 3C,D). The diminished *necab2* transcript in the mutant was presumably due to non-sense mediated RNA decay (Lykke-Andersen and Jensen, 2015). Since *necab1, necab2*, and *necab3* all share conserved domains with overlapping expression, it was evaluated whether the upregulation of *necab1* and *necab3* might compensate for the loss of *necab2*. A statistically significant increase was detected for *necab1* (*p* = 0.0255) but not *necab3* (*p* = 0.4238) transcripts (Figure 3D), suggesting potentially minimal compensation effects from these genes.

For gain-of-function studies, we generated overexpression lines of the two *necab2* isoforms (*necab2-001 and 201*) under the control of *hsp70*, a heat shock promoter. EGFP linked to Necab2 *via* the viral E2A element was used as a marker for screening (Figure 3E). The validity of Tg(*hsp:necab2-001:E2A:EGFP*) and Tg(*hsp:necab2-201:E2A:EGFP*) was verified by fluorescent imaging and semi-quantitative RT-PCR after 1 h 37°C heat-shock at 1 dpf (Figures 3F,G).

### The *necab2* Mutant Exhibits Decreased Baseline Locomotor Activity, Reduced Thigmotaxis Behavior, and Impaired Social Interactions

**FIGURE 4:**
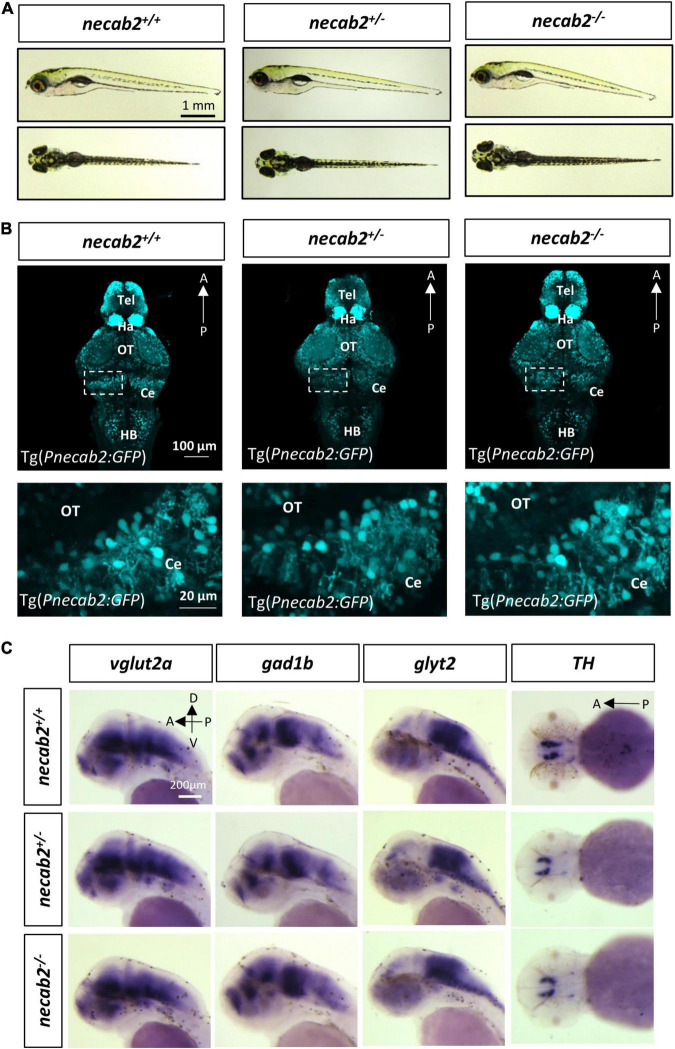
The *necab2^–/–^* larvae display grossly normal morphology and neuronal marker expression. **(A)** The *necab2*^+/–^ and *necab2*^–/^*^–^* larvae exhibited grossly normal appearance compared to wildtype siblings. The 7 dpf larvae derived from the heterozygous *necab2*^+/–^ mating were imaged blindly and followed by genotyping. Scale bar = 1 mm. **(B)** The *necab2*^+/–^ and *necab2*^–/^*^–^* larvae exhibited normal neural cytoarchitecture compared to wildtype siblings. A total of 7 dpf larvae were derived from heterozygous *necab2*^+/–^ mating in the Tg(*Pnecab2:EGFP*) background. Genotyping using the dissected tail was done after fixation for anti-EGFP immunofluorescence staining. Scale bar = 100 μm. The region in the dashed white box was shown at higher magnification below. Scale bar = 20 μm. **(C)** The *necab2*^+/–^ and *necab2*^–/^*^–^* larvae exhibit grossly normal composition of neuronal types. Spatial analysis of the glutamatergic (*vglut2a*), GABAergic (*gad1b*), glycinergic (*glyt2*), and dopaminergic (*TH*) neurons by whole-mount mRNA *in situ* hybridization was conducted blindly in the embryos derived from in-cross of *necab2*^+/–^ followed by genotyping. For each group, a total of 20 larvae were processed for *in situ* hybridization in order to obtain at least three homozygous larvae respectively. Scale bar = 200 μm. dpf, day post-fertilization; Tel, telencephalon; Di, diencephalon; Ce, cerebellum; re, retina; Ha, habenula; OT, optic tectum; MHB, midbrain-hindbrain boundary; HB, hindbrain; lens, crystalline lens.

**FIGURE 5:**
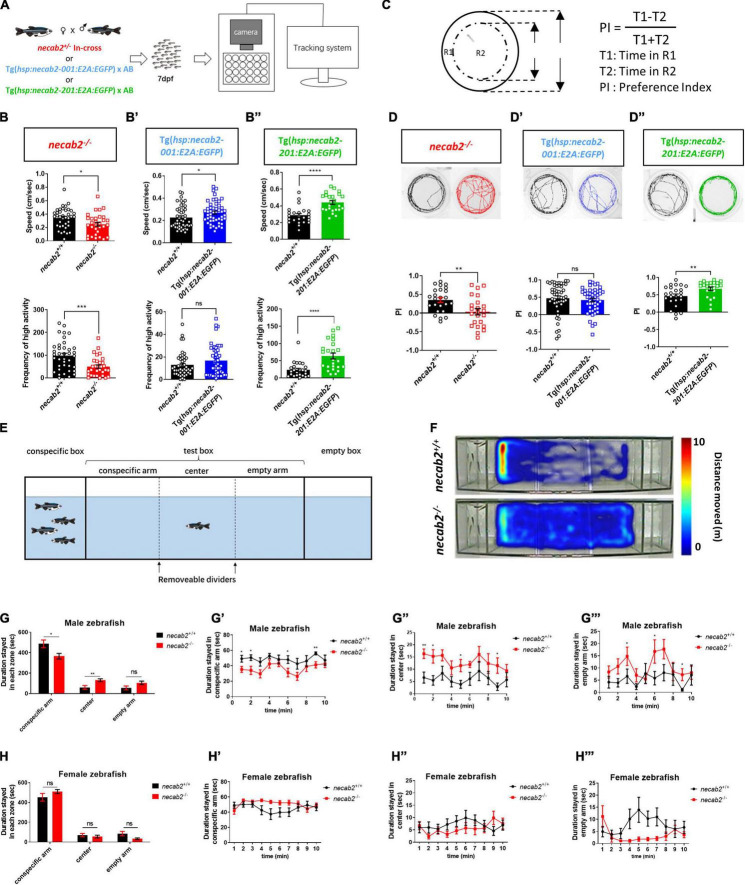
Necab2 is necessary and sufficient to promote psychomotor, thigmotaxis, and social interaction. **(A)** Workflow and experimental setup. To control for potential differences in the genetic background, tracking experiments were performed blindly on the progeny from heterozygous *necab2*^+/–^ mating. Genotyping was done after behavioral tracking by PCR. **(B–B″)** *necab2*^–/^*^–^* (in red, *n* = 31, with sibling control *n* = 38) larvae exhibited locomotor hypo-activity while Tg(*hsp:necab2-001:E2A:EGFP*) larvae (in blue, *n* = 48, with sibling control *n* = 45) and Tg(*hsp:necab2-201:E2A:EGFP*) larvae (in green, *n* = 24, with sibling control *n* = 24) yielded the opposite. (ns *p* ≥ 0.05, *p < 0.05, ***p < 0.001, ****p < 0.0001, Student’s *t*-test or Mann-Whitney *U* test). **(C)** Diagram of the thigmotaxis behavior test. The equation for calculating the preference index (PI) was shown at right. PI was calculated as the subtraction of the locomotion duration in the outer region (R1) and inner region (R2) divided by the total duration. R1 and R2 were of the same area. **(D–D″)** The *necab2*^–/^*^–^* (in red, *n* = 23, with sibling control *n* = 24) larvae exhibited decreased preference for the periphery region while Tg(*hsp:necab2-201:E2A:EGFP*) larvae (in green, *n* = 23, with sibling control *n* = 24) yields the opposite. Tg(*hsp:necab2-001:E2A:EGFP*) larvae (in blue, *n* = 48, with sibling control *n* = 47) showed no difference. (ns *p* ≥ 0.05, **p < 0.01, Student’s *t*-test or Mann-Whitney *U* test). **(E)** Diagram of the three-chamber social behavior test. Four unfamiliar conspecifics with mixed-sex were placed in the conspecific box while the box of the same size on the opposite end was empty. The test box in between was evenly divided into three zones—conspecific arm, center, and empty arm. The fish in the test was placed in the center bordered by two opaque dividers for one minute as adaption. **(F)** Representative heat maps of the duration stayed in each zone in the *necab2*^–/^*^–^* zebrafish and its wild-type sibling. **(G–G″′)** Duration stayed in each zone of the male progeny of in-crossed *necab2*^+/–^ fish at 3 months old. Columns showed total duration spent in each zone of *necab2*^+/+^ (*n* = 9) and *necab2*^–/^*^–^* (*n* = 9). Line diagrams showed the duration per minute spent in each zone. (ns *p* ≥ 0.05, *p < 0.05, **p < 0.01 Student’s *t*-test or Mann-Whitney *U* test). **(H–H″′)** Duration stayed in each zone of the female progeny of in-crossed *necab2*^+/–^ fish at 3 months old. Columns showed total duration spent in each zone of *necab2*^+/+^ (*n* = 9) and *necab2*^–/^*^–^* (*n* = 9). Line diagrams showed the duration per minute spent in each zone. (ns *p* ≥ 0.05, Student’s *t*-test or Mann-Whitney *U* test). The duration was not significantly different at each time point. Speed: average speed calculated by the computer in 15–30 min. Frequency of high activity: the times that more than 80% of the pixels from the zebrafish body moved per second.

Many aspects of development examined in the *necab2* mutant so far appeared normal: the grossly normal appearance (Figure 4A), normal neural development and cytoarchitecture (Figure 4B), and the balanced composition of neuronal subtypes (Figure 4C), as well as good fertility in adulthood (data not shown). To determine whether the *necab2* mutant displayed altered behaviors as those commonly observed in ASD patients, we recorded larval locomotion and anxiety-associated behaviors, adapted as markers of ASD behaviors in zebrafish (Kozol et al., 2015; Zimmermann et al., 2015; Li et al., 2019; Maphanga et al., 2020). To control for possible differences in genetic backgrounds, behavioral tracking was performed on the progeny derived from heterozygous *necab2*^+/–^ mating, followed by blind genotyping (Figure 5A). The *necab2* mutant larvae showed a decrease in baseline locomotor activity, measured by both the speed (*p* = 0.0136) and the frequency of high activity (*p* = 0.0007) (Figure 5B). Thigmotaxis (i.e., preference for edges) is used as a measure of anxiety in rodents (Treit and Fundytus, 1988; Simon et al., 1994). Larval zebrafish also display this anxiety-like behavior as young as 5 dpf (Schnörr et al., 2012; Lundegaard et al., 2015). The preference index (PI,% time in edge –% time in the center) (Figure 5C) was quantified, which revealed that the mutant larvae had significantly lower PI (*p* = 0.0480), indicating either a reduced fear-like response or decreased cognition needed for spatial navigation (Figure 5D). Strikingly, overexpression of *necab2-201* isoform in larval stage yielded behaviors opposite to that of the mutant (Figures 5B″,D″). Overexpression of the *necab2-001* isoform in larval stage had milder effects and at times did not reach significance (Figures 5B′,D′). Taken together, opposite behaviors in the loss-of-function and gain-of-function models suggest that *NECAB2* is necessary and sufficient for promoting locomotor and thigmotaxis behaviors.

Since the *NECAB2* loss-of-function was found in ASD patients, we wondered whether the *necab2* mutant might exhibit altered social behaviors as observed in many zebrafish autism models (Kim et al., 2017; Liu et al., 2018). For that, the three-chamber social preference test was used (Figure 5E; Lachlan et al., 1998). Wild-type adult zebrafish preferred to stay in the conspecific arm (Figure 5F). Similar to the larval assays above, behavior tracking was done blindly on the adult progeny derived from heterozygous *necab2*^+/–^ mating, followed by genotyping. Given the 4:1 male/female ratio observed in human autism (Schaafsma and Pfaff, 2014), data in different sexes were analyzed. We found that adult *necab2^–/–^* males spent significantly less time in the conspecific arm (*p* = 0.0400) and comparatively more time in the center arm (*p* = 0.0075) as well as the empty arm (*p* = 0.0530) than their wild-type counterparts in total and throughout the examination window (Figures 5G–G″′). In contrast to males, adult *necab2^–/–^* females manifested similar social preference compared to their wild-type counterparts (Figures 5H–H″′). Together, *necab2* deficiency impairs social interaction in adult zebrafish in a male-specific manner.

### Necab2 Had Potential Roles in Modulating the G-Protein Coupled Receptor Signaling

**FIGURE 6:**
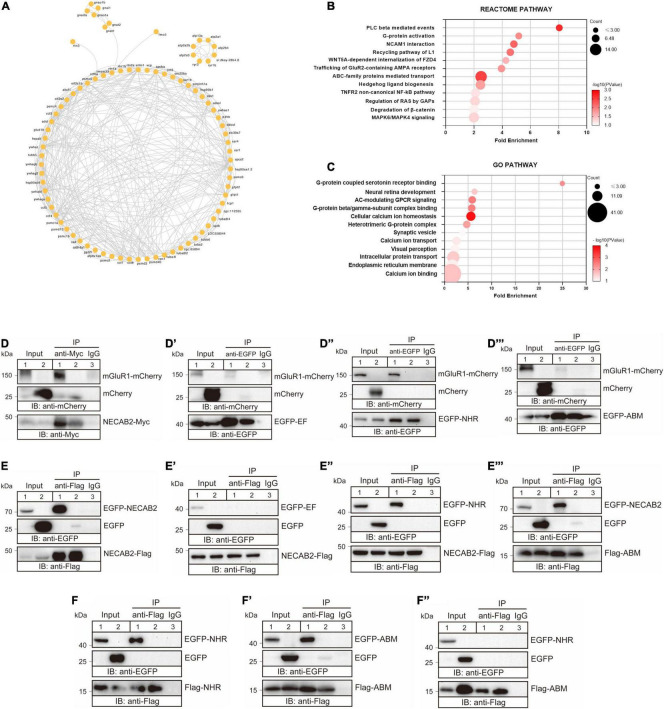
NECAB2 interacts with mGluR1 *in vitro*. **(A)** Overview of the protein interaction network derived from the Necab2 co-immunoprecipitation and subsequent mass spectrometry in the *necab2*^+/+^ and *necab2^–/–^* larvae. **(B)** REACTOME analysis revealed the significant biological processes detected in the *necab2*^+/+^ larvae compared to the *necab2^–/–^* larvae. **(C)** Gene Ontology (GO) analysis revealed the significant biological processes detected in the *necab2*^+/+^ larvae compared to the *necab2^–/–^* larvae. **(D–D″′)** NECAB2 interacts with mGluR1 through the NHR domain uncovered by co-immunoprecipitation analysis. The HEK293 cells were transiently transfected with mGluR1-mCherry plus NECAB2-Myc **(D)**, EGFP-EF **(D′)**, EGFP-NHR **(D″)**, or EGFP-ABM **(D″′)** for lane 1 and lane 3 respectively, and indicated vectors plus mCherry-vector for lane 2 and processed for immunoprecipitation using mouse anti-Myc antibody **(D)** or anti-EGFP antibody **(D–D″′)**, with normal mouse IgG for negative control in lane3. The crude extracts (Input) and immunoprecipitations (IP) were analyzed by SDS-PAGE and immunoblotted using the rabbit anti-mCherry antibody **(D–D″′)**, mouse anti-Myc antibody **(D)** or mouse anti-EGFP antibody **(D′–D″′)**. **(E–E″′)** NECAB2 has self-interaction through either NHR or ABM domain uncovered by co-immunoprecipitation analysis. The HEK293 cells were transiently transfected with NECAB2-Flag plus EGFP-NECAB2 **(E)**, NECAB2-Flag plus EGFP-EF **(E′)**, NECAB2-Flag plus EGFP-NHR **(E″)**, or NECAB2-Flag plus EGFP-ABM **(E″′)** for lane 1 and lane 3, and the indicated vectors plus EGFP-vector for lane 2 and processed for immunoprecipitation using mouse anti-Flag antibody, with normal mouse IgG for negative control in lane 3. The crude extracts (Input) and immunoprecipitations (IP) were analyzed by SDS-PAGE and immunoblotted using mouse anti-EGFP antibody **(E–E″′)** or mouse anti-Flag antibody **(E–E″′)**. **(F–F″)** Co-immunoprecipitation analysis of NHR and ABM domains showed self-interaction but no cross-interaction of the two domains. The HEK293 cells were transiently transfected with Flag-NHR plus EGFP-NHR **(F)**, Flag-ABM plus EGFP-ABM **(F′)**, and Flag-ABM plus EGFP-NHR **(F″)** for lane 1 and lane 3, and indicated vectors plus EGFP-vector for lane 2 and processed for immunoprecipitation using the mouse anti-Flag antibody, with normal mouse IgG for negative control in lane3. The crude extracts (Input) and immunoprecipitations (IP) were analyzed by SDS-PAGE and immunoblotted mouse anti-Flag antibody **(F–F″)** or mouse anti-EGFP antibody **(F–F″)**.

Thus far, our data showed that *necab2*, which was expressed in synaptic terminals of glutamatergic and GABAergic neuronal subtypes, played a critical role in regulating psychomotor behaviors in larval zebrafish and social interactions in adult male zebrafish. To further probe its molecular and biochemical actions, we performed co-immunoprecipitation (Co-IP) using the anti-Necab2 antibody in both *necab2*^+/+^ and *necab2^–/–^* zebrafish larvae followed by mass spectrometry (MS) to identify the Necab2 interactome *in vivo*. Coomassie blue staining on the Co-IP samples, followed by western blotting (WB), confirmed the presence of two Necab2 isoforms and the specificity of the antibody (Supplementary Figure 5). Proteins co-immunoprecipitated in the *necab2^–/–^* zebrafish larvae were presumed to be non-specific and therefore served as a control. A number of Necab2-interacting proteins were discovered using this approach (Figure 6A). Pathway enrichment analysis identified significant interactions of Necab2 with proteins in several biological processes, including the protein lipase C-beta (PLCβ)-mediated events (R-DRE-112043), G-protein activation (R-DRE-202040), G-protein coupled serotonin receptor binding (GO:0031821), and G-protein beta/gamma-subunit complex binding (GO:0031683) (Figures 6B,C). These results indicate that Necab2 is involved in modulating signal transduction cascades of G-protein coupled receptors (GPCRs).

### Necab2 Interacts With mGluR1 *in vitro*

Several studies have shown the emerging role of mGluR1, which is a member of the type I metabotropic glutamate receptors, in the development of ASD (Martin et al., 2010; Ribeiro et al., 2010; Tsai et al., 2012). Considering its abundance in the cerebellum and potential role as a GPCR modulator, we wondered whether Necab2 interacts with mGluR1. There are two paralogous loci of mGluR1, *grm1a* and *grm1b*, in the zebrafish genome (Haug et al., 2013). Due to the lack of full-length cDNA clones for the zebrafish genes, we performed co-IP using the mouse mGluR1 (*Grm1*) cDNA clone in cultured mammalian cells. In HEK293T cells, we detected an interaction between rodent NECAB2 and mGluR1 proteins (Figure 6D). To determine which NECAB2 domain(s) are responsible for mGluR1 binding, truncated forms of NECAB2 were combined with mGluR1 for transfecting HEK293T cells. Co-IP revealed that NECAB2 interacted with mGluR1 through the NHR domain (Figure 6D″), instead of the EF-hand domain (Figure 6D′) or the ABM domain (Figure 6D″′). To further explore the roles of NECAB2 domains, the Co-IP experiments between the NECAB2-Flag and EGFP-NECAB2, or the segmented domains with different tags, were performed. NECAB2s were able to interact with each other (Figure 6E), through either the NHR domain (Figures 6E″,F) or the ABM domain (Figures 6E″′,F′). The EF-hand domain did not participate in the self-interaction (Figure 6E′) and the NHR domain was not able to cross interact with the ABM domain (Figure 6F″). In summary, NECAB2 interacts with mGluR1 through the NHR domain *in vitro*. The NHR domain and the ABM domain probably play a role in the self-interaction of NECAB2.

### Necab2 Interacts With mGluR1 *in vivo* and Is Sufficient to Promote mGluR1 Signaling

**FIGURE 7:**
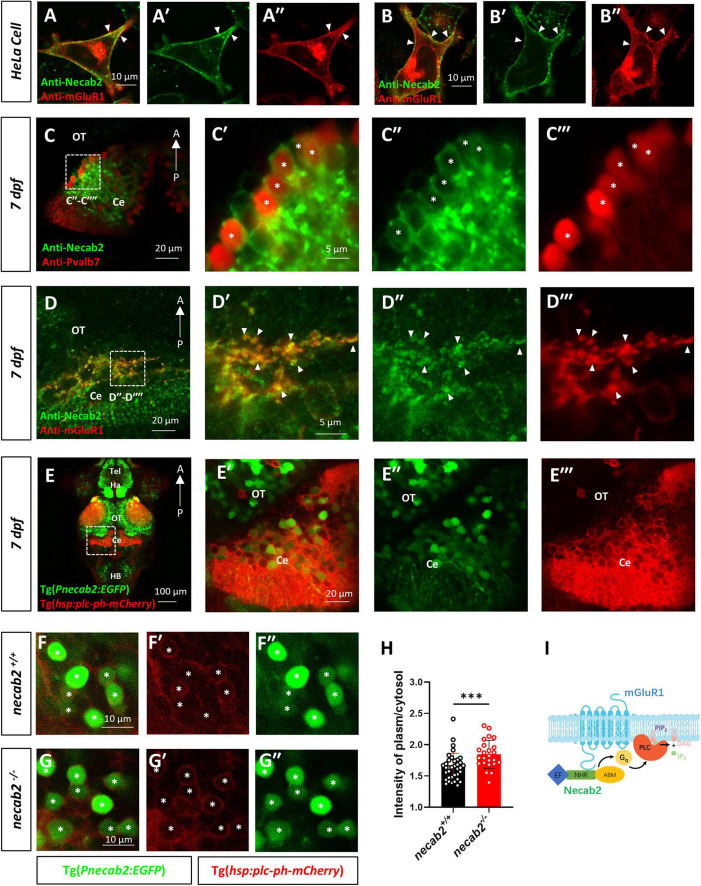
Necab2 co-localizes with mGluR1 and promotes PIP2-PLC-PKC signaling. **(A–A″,B–B″)** NECAB2 and mGluR1 colocalize at the cell membrane of HeLa cells (arrowheads). The HeLa cells were transiently transfected with EGFP-NECAB2 and mGluR1-mCherry and live imaging was conducted with the confocal microscope. Scale bar = 10 μm. **(C–C″′)** Co-immunofluorescent staining of anti-Necab2 with anti-Pvalb7 in 7 dpf larvae showed that Necab2 was expressed in the Purkinje cells in the cerebellum (asterisks). Scale bar = 20 μm **(C)**. The region in the dashed white box **(C)** is shown at higher magnification on the right **(C′–C″′)**. Scale bar = 5 μm. **(D–D″′)** NECAB2 and mGluR1 co-localized in the cerebellum of the zebrafish larvae (arrowheads). The *necab2*^+/+^ larvae at 7 dpf were processed for anti-Necab2 and anti-mGluR1 immunofluorescence staining following fixation. Scale bar = 20 μm **(D)**. The region in the dashed white box **(D)** was shown at higher magnification on the right **(D′–D″′)**. Scale bar = 5 μm. **(E–E″′)** Live imaging of *necab2*^+/+^ larvae at 7 dpf in Tg(*hsp70:plc-ph-mCherry*) and Tg(*Pnecab2:EGFP*) background by confocal microscope. Z-stack, Maximum intensity projection. Scale bar = 100 μm **(E′)**. The cerebral region (in the dashed white box) was shown at higher magnification on the right **(E′–E″″)**. Scale bar = 20 μm. **(F–F″,G–G″)** The *necab2*^–/^*^–^* larvae accumulated more PLC-PH-mCherry at the cell membrane of the necab2-expressing cells (asterisks) than that of the *necab2*^+/+^ larvae. Genotyping of the 7 dpf larvae derived from heterozygous *necab2*^+/–^ in the Tg (*Pnecab2:EGFP*) and Tg(*hsp70:plc-ph-mCherry*) background mating was done after live imaging with the confocal microscope. Scale bar = 10 μm. **(H)** The quantification of the PLC-PH-mCherry intensity in the *necab2*^–/^*^–^* larvae (*n* = 6) and *necab2*^+/+^ (*n* = 4) larvae by calculating the fluorescent intensity of plasma/cytosol. Two different sections per larva and three independent necab2-expressing cells per section were analyzed. (***p < 0.001, Mann–Whitney U test). **(I)** The summary of Necab2 interacting with mGluR1. Necab2 bound mGluR1 through the NHR domain. Necab2 was likely to facilitate the mGluR1 mediated PLC-PIP2-PKC signaling. dpf, day post-fertilization; Tel, telencephalon; Ha, habenula; OT, optic tectum; Ce, cerebellum; HB, hindbrain.

To explore whether Necab2 and mGluR1 interact *in vivo*, whole-mount immunofluorescent staining and confocal imaging were performed in the larval zebrafish cerebellar neurons. Since Grm1a, but not Grm1b has been identified in the cerebellum of zebrafish (Haug et al., 2013), a polyclonal antibody was raised in the rat against Grm1a, yielding good specificity (Supplementary Figure 2C). We first performed co-localization analysis of mouse Necab2 and Grm1a transfected into Hela cells. Strong co-localization of NECAB2 and mGluR1 was observed especially at the cell membrane (Figures 7A–A″,B–B″), which suggests possible membrane association of Necab2. We next identified *necab2*-expressing neuronal subtypes in the cerebellum by performing confocal live imaging in Tg(*Pnecab2:EGFP*) and Tg(*gad1b:DsRed*) backgrounds. The results indicated that necab2 is expressed both in the GABAergic Purkinje neurons or interneurons (Supplementary Figures 6A–A″′) and the glutamatergic granule neurons (Supplementary Figures 6B–B″′,C–C″′). More specifically, Necab2 was abundant in Pvalb7-positive Purkinje neurons (Figures 7C–C″′) and colocalized with mGluR1 (Figures 7D–D″′). Together, these results indicate that Necab2 is associated with mGluR1 at the membrane of GABAergic Purkinje cells.

To explore whether the interaction between Necab2 and mGluR1 has functional relevance, we measured the downstream effectors of mGluR1 signaling. Stimulation of type I mGluR1 activates phospholipase C (PLC), which hydrolyzes phosphatidylinositol 4,5-bisphosphate (PIP2) to produce inositol trisphosphate (InsP3) and diacylglycerol (DAG), thereby increasing intracellular calcium concentration, stimulating protein kinase C (PKC), and generating slow excitatory postsynaptic currents (Venkatachalam and Montell, 2007). Measurement of PIP2 hydrolysis *in vivo* has been developed by injecting mRNAs encoding the PH domain of PLC (*that is*, the PIP2 binding domain) into zebrafish embryos (Gong et al., 2017). By fusing with mCherry, the red fluorescent signal of PLC-PH-mCherry protein indicates the abundance of PIP2 in cell membrane. By generating the transgenic line Tg(*hsp:plc-ph:mCherry*), we measured the PLC-PH-mCherry signal in *necab2^–/–^* and WT siblings at 7dpf (Figure 7E and Supplementary Figure 7). The PLC-PH-mCherry protein was abundant in the cerebellum showing strong membrane association indicating a high content of PIP2 in this region (Figures 7E–7E″′). Confocal live imaging was performed on the progeny derived from the heterozygous *necab2*^+/–^ mating, followed by blind genotyping. Compared to their *necab2*^+/+^ siblings (Figures 7F–F″), *necab2^–/–^* larvae accumulated more PLC-PH-mCherry at the cell membrane of *necab2*-expressing cells identified by Tg(*Pnecab2:EGFP*) (Figures 7G–G″). Semi-quantitative measurement of the plasma/cytosol mCherry intensity suggested more PIP2 and reduced PLC-PIP2-PKC signaling in the *necab2^–/–^* larvae (*p* = 0.0003) (Figure 7H). In support of Necab2’s role in regulating mGluR1 signaling rather than its synthesis or localization, we found that the expression of mGluR1 was unchanged in *necab2*^–/^*^–^* (Supplementary Figures 6D,E).

Thus, our data suggest that Necab2 interacts with mGluR1 to promote its signaling in cerebellar Purkinje and possible other neuronal types. Necab2 binds to mGluR1 through the NHR domain. The interaction facilitates the PIP2 hydrolysis and probable mGluR1 mediated downstream signal transductions to mediate neuronal activation (Figure 7I).

## Discussion

Genotypic and phenotypic complexities in ASD patients have been well recognized (Vorstman et al., 2017). Although the number of ASD candidate loci has expanded significantly, functional studies significantly lag behind. *NECAB2* is a potential risk gene for ASDs. A segmental deletion in the *NECAB2* gene spanning the human chromosome 16q23.3-q24.1 has been reported in a proband with idiopathic ASD (Sakai et al., 2011). Two individuals with deletions in chromosome 16q23.3 encompass *NECAB2* and three additional genes have also been reported (Itsara et al., 2010; Sanders et al., 2011; Supplementary Table 1). Meanwhile, missense mutations in *NECAB1* are associated with developmental language disorders, one of the frequent comorbidities in autism (Kornilov et al., 2016). Despite the roles in pro-nociceptive pain signaling in the spinal cord (Zhang et al., 2014, 2018; Ma et al., 2021), the potential role of *NECAB2* in the brain remains largely elusive. In this study, we have reported the *in vivo* brain distribution of *necab2* at both tissue, cellular, and subcellular levels. We have also investigated the morphological and behavioral phenotypes in both the loss-of-function and gain-of-function models of zebrafish. Furthermore, *necab2* was found to interact with mGluR1 both *in vitro* and *in vivo* and such interaction served to modulate mGluR1 signaling *via* the PLC-PIP2-PKC cascade in the cerebellum.

The *necab2* knock-out zebrafish generated *via* CRISPR developed normally but showed altered psychomotor and social behaviors. The double-blind, high-throughput behavior analysis in larvae demonstrated that the *necab2*^–/^*^–^* larvae were hypoactive, less anxious, or cognitively compromised in spatial navigation, while the *necab2*-overexpressing larvae demonstrated behavioral phenotypes that were opposite to the loss-of-function model. The impaired locomotor activity was commonly observed in other zebrafish models of autism (Bailey et al., 2016; Lee et al., 2018; Liu et al., 2018). The reduction in the thigmotaxis was also reported in some ASD models, such as the *shank3b* mutant (Liu et al., 2018). Therefore, the amenability to these behaviors as high-throughput assays for small molecule drug screening is a tremendous advantage of the zebrafish model. Importantly, the social deficits observed in the *necab2* knock-out zebrafish were reminiscent of that in ASD patients, reinforcing the notion that disruption of *necab2* causes ASD-relevant symptoms. It is interesting to note that only male zebrafish exhibit impaired social interaction, which correlates well with the high male/female ratio in ASD patients and lower penetrance of ASD in female patients (Lai et al., 2015). One possible explanation is that estrogen has a protective effect against ASD since estrogen was found to ameliorate ASD-like behaviors in zebrafish larva (Hoffman et al., 2016). Meanwhile, the interaction between estrogen receptors and mGluR1 could facilitate the glutamatergic neurotransmission at parallel fiber-Purkinje cell synapses (Hedges et al., 2018). However, the exact mechanism underlying the male-specific social defects in *necab2^–/–^* zebrafish warrants further investigations. Additional behavioral assays are certainly needed to further explore other ASD-related behaviors in the *necab2* mutants, such as learning and cognitive impairments.

It is interesting to note that Necab2 was abundant in the GABAergic cerebellar Purkinje cells. The cerebellum not only coordinates a person’s motor ability but also plays an important role in language, emotion, and cognition (Wang et al., 2014). The cerebellar role in autism has been highlighted in neuroanatomical (Amaral et al., 2008), neuroimaging (D’Mello et al., 2015), and gene-expression studies (Menashe et al., 2013). The decrease in the number and activity of Purkinje cells was evident in either ASD patients (Ritvo et al., 1986) or gene knock-out models (Tsai et al., 2012). Subcellularly, Necab2 was not confined to cell bodies but was abundantly detected in peri-synaptic structures, leading to the hypothesis that Necab2 might be involved in synaptic signal transduction, the dysfunction of which has been regarded as one of the key features in ASD pathophysiology (Ebrahimi-Fakhari and Sahin, 2015; Masini et al., 2020). Combined with ASD-like behaviors in the *necab2^–/–^* model, our finding relating to cerebellar Purkinje cells provides an excellent cellular context to further probe Necab2’s role in regulating synaptic physiology.

Although NECAB2 has been identified about 20 years ago (Sugita and Sudhof, 2000), the understanding of its molecular role and biochemical function is still limited. One of the most important aspects of our findings was the establishment of a physical interaction between Necab2 and mGluR1. Defects in the type I mGluRs and their downstream signaling events have been considered to be one of the major causes of autism (Zoghbi and Bear, 2012). In contrast to the ionotropic glutamate receptors that mediate fast excitatory synaptic transmission, mGluRs modulate neuronal excitability and synaptic plasticity through a variety of intracellular second messengers (Niswender and Conn, 2010). mGluR1 couples to G_α_ q/G11 to activate the PLC-β, which cleaves PIP2 into DAG and InsP3. InsP3 then binds to its corresponding receptor on the ER membrane to increase the cytoplasmic Ca^2+^ ion levels (Katan and Cockcroft, 2020). Both Ca^2+^ and DAG stimulate PKC to phosphorylate AMPA, which leads to internalization and long-term depression. Our study found that the *necab2* knock-out accumulated PIP2 in the cell membrane, indicating a reduced PLC-β activity and in turn downstream PKC activity. This observation suggests that Necab2 facilitates the signaling transduction of mGluR1. Our study further showed that Necab2 bound mGluR1 through NHR domain. The NHR domain and ABM domain probably involve in the self-interaction of NECAB2, however, the exact roles warrant further studies. Previously, NECAB2 was found to interact with another type I mGluR member, mGluR5, and potentiate its subsequent IP3 accumulation (Canela et al., 2009). Though, the exact significance of the macromolecular complexes composed of mGluR1 and Necab2 remained to be clarified, we speculated that the interaction among mGluR1, Necab2, and other potential protein, similar to the interaction between mGluR5 and NECAB2, probably promote subsequent signaling by ensuring the proximity of G-proteins or PLCβ with mGluR1. However, testifying the above hypothesis demands additional experiments. For one thing, it will be interesting to find out whether an increase in cytoplasmic Ca^2+^ has a regulatory role over the interaction between Necab2 and mGluR1 *in vivo*.

## Limitation

The significance of our study is limited by the following points: First, the expression of necab2 was only profiled in the larva stage. It remains to be investigated whether the expression pattern differs in the adult brain. Second, the interaction between Necab2 and mGluR1 is validated in both transfected cells and intact brains, but the evidence for Necab2 modulating mGluR1 signaling is still limited. Future biochemical experiments should be performed in the zebrafish model with a focus on how necab2 promotes mGluR1 signaling through the interaction and its downstream effects. Third, Necab2 has a relatively broad expression spectrum in the nervous system, and the role of Necab2 in other types of neurons should not be neglected. Another potential drawback of our study is the absence of the rescue experiment. Finally, the homology between zebrafish and humans should be cautioned especially when interpreting the behaviors.

## Conclusion

In conclusion, this study has shed new light on the *in vivo* function of *necab2*, a locus associated with ASD risk. We characterized the phenotypes of *necab2* loss-of-function and gain-of-function in the zebrafish models and provided direct evidence for the interaction between Necab2 and mGluR1 both *in vitro* and *in vivo*. The domains involved in the interaction were also mapped. Manifestation of ASD-like behaviors in the *necab2* loss of function model further helps establish *necab2* as a disease-causing locus and provides a new avenue for mechanistic studies and therapeutic discovery.

## Materials and Methods

### Protein Diagrams and Polygenetic Tree

Protein diagrams of the NECAB2 orthologs in *H. sapiens*, *M. musculus*, and *D. rerio* were generated using IBS 1.0 from published NCBI protein sequences (Liu et al., 2015). Phylogenetic tree of the evolutionary relationship of NECAB2 proteins and evolutionary analyses were conducted using the MEGA7 software (Kumar et al., 2016).

### Zebrafish Maintenance and Strains

The zebrafish were raised and maintained in accordance with a standard protocol (Westerfield, 2000). All wildtype zebrafish used in the present study are AB strain. The *necab2* mutant was generated using the CRISPR/Cas9 technology as described previously (Hwang et al., 2013). The *necab2* sgRNA (AATTTAATACGACTCACTATAGGGCTTCGCGCCGGAGCC TCGAAGTTTAAGAGCTATGCT) was transcribed *in vitro* from the DNA templates of the amplified PCR products of the pMD19-T-gRNA vector (CZRC, China). The Cas9 mRNA was generated *in vitro* by transcription with a linearized plasmid pT3TS-nlszCas9-nls (CZRC, China). The *Cas9* mRNA and *necab2* sgRNA were co-injected into the one-cell stage embryos. The positive founders were mated with the wild-type fish to obtain F1 generation. The F1 heterozygous zebrafish with identical frameshift mutations were intercrossed to generate an F2 homozygous mutant.

The Tg (*hsp:necab2-001:E2A: EGFP*) and Tg (*hsp:necab2-201:E2A: EGFP*) were generated *via* the Tol2 transposition system as described previously (Kawakami et al., 2016). Briefly, the *necab2-001* or *necab2-201* cDNA fragment was cloned into the pT2KhspGFF. The transposase mRNA was generated by *in vitro* transcription with a linearized plasmid pCS-zTP. A Tol2-donor plasmid DNA and the transposase mRNA were introduced into the zebrafish fertilized eggs by microinjection. The positive founders were mated with wild-type fish to obtain F1 generation. The larvae were raised at 29°C and heat-shocked at 1 dpf for 1.5 h before screening or experiments. The Tg (*hsp:plc-ph:mCherry*) was produced by cloning the PH domain of the PLC fused with mCherry from pXT7-PLCδ1a-PH-mCherry into the pT2Khsp. The plasmid was obtained from the laboratory of the Shunji Jia at Tsinghua University.

The reporter line, Tg (*Pnecab2:EGFP*) was generated similarly. The full-length upstream promoter region was cloned into the pT2KhspGFF after being verified by the Sanger sequencing. The fragment length we cloned is 7848 bp located in Chromosome 18: 21,401,324-21,409,171 (NC_007129.7), which contains the potential regulating sequences spanning from the 3′UTR of the upstream gene (*hydin*) to the intron between exon 1 and exon 2 of *necab2*.

The Tg(*vglut2a:DsRed*), Tg(*gad1b: DsRed*), and Tg(*glyt2:DsRed*) lines were obtained from the National BioResource Project, Zebrafish, Core Institution (Saitama, Japan), and were deposited by the National Institute of Natural Science (Dr. Shin-ichi Higashijima) (Higashijima et al., 2004).

### Behavior Profiling

The behavioral assays were conducted as described previously (Bai et al., 2016). Behavioral testing was performed in the *DanioVision* system at a constant water-bath temperature of 29°C and at time-points between 11:00 and 16:00 h to avoid unexpected factors that might contribute to locomotion. High activity is defined as more than 80% change of the pixels of the targeted larva during one frame, which is defined and measured by the built-in algorithm. All the behaviors were tracked at 60 frames per second. To control for potential differences in genetic backgrounds, all the tracking experiments for mutants were performed blindly on the progeny of heterozygous mating. After tracking, the genomic DNA was isolated from individual larvae and genotyping was done by PCR. Behavior profiling was done in 7 dpf larvae and 3-month-old zebrafish, respectively. Larvae of overexpression lines were derived from Tg(*hsp:necab2-001:E2A:EGFP*) and Tg(*hsp:necab2-201:E2A:EGFP*) mating with wildtype respectively, and were raised at 29°C and tested after 1.5 h at 37°C heat-shock and 1 h at 29°C for accommodation. The genotypes were identified by selecting the fluorescent larvae under the microscope after behavior tracking, while the larvae without fluorescent were identified as sibling controls.

### *In vivo* PIP2 Assay and Confocal Live Imaging

The PIP2 assay was performed according to the previously study with some modifications (Haug et al., 2013). Confocal imaging and fluorescent quantitative analysis were performed blindly on the progeny of heterozygous *necab2*^+/–^ mating. The larvae were mounted in low melt agarose and live-imaging was performed under the Zeiss LSM880 confocal microscope (Zeiss). Images were processed using the ZEN2012 software (Zeiss) and the quantification analysis was performed to measure the intensity of the plasma/cytosol using the ImageJ (64-bit Java 1.8.0_172). Specifically, the area and fluorescent intensity of the whole cell and the cytoplasm were outlined and measured manually. The intensity of the plasma membrane was calculated by subtraction: the plasma fluorescent intensity = (mean fluorescent intensity of whole cell × area of whole cell − mean fluorescent intensity of cytosol × area of cytosol)/(area of whole cell − area of cytosol). The plasma/cytosol intensity = mean fluorescent intensity of plasma/mean fluorescent intensity of cytosol. Subsequently, genomic DNA was isolated from individual larvae and genotyped *via* PCR.

### *In situ* Hybridization and Immunohistochemistry

*In situ* hybridization was performed according to the previously described method with some modifications (Peng et al., 2009). Briefly, larvae were fixed in 4% PFA at different ages after PTU treatment to inhibit pigment synthesis. Embryos were hybridized with the designed digoxin-labeled RNA probe. After staining, embryos were washed in PBST and cleared in benzyl benzoate/benzyl alcohol for imaging.

Whole-mount antibody staining of dissected embryos was performed as previously described, with some modifications (Peng et al., 2009). Briefly, larvae were fixed in 4% PFA at different ages following PTU incubation. After fixation, embryos were washed in PBS, dissected, blocked for at least 1 h at room temperature, and incubated in the primary antibodies overnight at 4°C. Embryos were washed 4–6 times in PBT and incubated in secondary antibodies overnight at 4°C. Afterward, embryos were washed 4–6 times at room temperature and mounted for imaging in low melt agarose (0.8–1%). The tubes containing embryos were rotated at each step.

The primary antibodies used in this study were as follows: The polyclonal rabbit anti-Necab2 antibodies were raised against the full-length zebrafish Necab2 protein (1 aa–428 aa) in association with the GeneCreate Biological Engineering Co., Ltd., in Wuhan, China, and used in 1:500 dilution. The polyclonal rat anti-GRM1 antibodies were raised against the C-terminal zebrafish grm1a protein (GDGKPAPCQSNSILNMFRRKKNNNNATGST NPNGKSVSWSESGARPQGRGSSVFHRLSVHVRRQAVGQSQ TAVIRPLTNASQPPESEYGAGLNTAPNGSHPDDKDLYNLGEG HDGGPQHPTAQEGGLPPSY) in collaboration with Dia-An Biotech, Inc., in Wuhan, China, and used in a 1:500 dilution. Other primary antibodies included: the mouse IgG1 anti-SV2 (1:500 dilution, DSHB, AB_2315387), mouse anti-pan-MAGUK (1:500 dilution, Antibodies Inc., AB_10673115), chicken anti-EGFP (1:500 dilution, Aves Labs, AB_2307313), and mouse anti-Pvalb7 (1:1,500 dilution, MYBioSource, MBS1321475).

The secondary antibodies used in this study were as follows: goat anti-rabbit 647 (1:1,000 dilution, abcam, ab150079), Anti-rat 568 (1:1,000 dilution, abcam, ab175476), and goat anti-Chicken 568 (1:1000 dilution, abcam, ab175477).

### Immunoprecipitation and Immunoblotting

For immunoprecipitation, HEK293 cells were transfected using the pCMV6 plasmids, into which cDNAs of truncated domains of NECAB2 and mGluR1 from mouse plus indicated tags were cloned, and Lipofectamine 2000 (Invitrogen) was used. The transiently-transfected HEK293 cells or zebrafish tissues were solubilized in the NETT lysis buffer (50 mM Tris-HCl, 150 mM NaCl, 0.1 mM EDTA, and 1% Triton X-100) following ultrasonic crushing. After centrifugation, supernatants were incubated overnight at 4°C with the mouse anti-Flag antibody (1 μg, Sigma, F1804), mouse anti-Myc antibody (1 μg, MBL, M192-3), mouse anti-EGFP antibody (1 μg, Abmart, M20004S), rabbit anti-Necab2 antibody (1 μg) or normal mouse IgG (1 μg, EMD Millipore Corporation, M12-371). Then, the protein A/G agarose beads (Santa Cruz Biotechnology) were added and rotated at 4°C for another 4 h. The beads were then washed by NETT buffer 5 times and finally boiled in the loading buffer for SDS-PAGE.

For immunoblotting, samples were fractionated on SDS polyacrylamide gels and transferred to PVDF membranes (Millipore). Afterward, membranes were immunoblotted with the mouse anti-Flag antibody (1:5,000 dilution, Sigma, F1804), mouse anti-Myc antibody (1:5,000 dilution, MBL, M192-3), mouse anti-EGFP antibody (1:5,000 dilution, Abmart, M20004S), rabbit anti-mCherry antibody (1:5,000 dilution, Abcam, ab183628), or rabbit anti-Necab2 antibody (1:5,000 dilution) followed by incubation in the correspondent secondary antibody (1:5,000 dilution, HRP-conjugated goat anti-mouse/rabbit IgG, CWbio, CW0102S/CW0103S). The immunoreactive bands were developed using an efficient chemiluminescence kit (Bio-Rad).

### Mass Spectrometry

The PAGE-resolved proteins were identified by mass spectroscopy using the LTQ Orbitrap Elite (Thermo Fisher Scientific) from the State Key Laboratory of Genetic Engineering of the School of Life Sciences, Fudan University, Shanghai, China. The protein-protein interaction network was based on STRING (Search Tool for the Retrieval of Interacting Genes/Proteins, available online at website: www.string-db.org) and repainted by the RStudio. The pathway analysis was based on DAVID (the Database for Annotation, Visualization, and Integrated Discovery) Bioinformatics Resources using the Reactome V62 and Gene Ontology. All of the non-human identifiers were converted to their human equivalents.

### Statistical Analysis

Statistical analyses were performed using SPSS version 25.0 (64-bit edition, IBM Corp., Armonk, NY, United States). The normality of the data was confirmed by the Shapiro-Wilk test and the quantitative variables were presented as mean ± standard error and median (quartiles) where appropriate. The descriptive variables were shown in the counts or proportions. The student’s *t*-test or Mann–Whitney U test was applied as appropriate for comparing the two independent groups. The one-way analysis of variance or Kruskal–Wallis test was used to compare the three or more independent groups. All the analyses were two-tailed, and the statistical significance was set as a *p*-value of < 0.05. The results of the two-sided tests were considered significant at *p* < 0.05.

## Data Availability Statement

The datasets presented in this study can be found in online repositories. The names of the repository/repositories and accession number(s) can be found in the article/Supplementary Material.

## Ethics Statement

The animal study was reviewed and approved by Human Research Ethics Committee of Fudan University.

## Author Contributions

ZC, JG, and HL constructed the zebrafish models. CT and HL performed the immunostaining. HL performed the behavior profiling. YW and HL carried out the western-blot and Co-IP. ZC and XL performed the confocal imaging. ZC and SG wrote the first draft of the manuscript. KH and KJ critically reviewed the manuscript. SG and YP supervised the project. All authors contributed to the study conception and design and read and approved the final manuscript.

## Conflict of Interest

The authors declare that the research was conducted in the absence of any commercial or financial relationships that could be construed as a potential conflict of interest.

## Publisher’s Note

All claims expressed in this article are solely those of the authors and do not necessarily represent those of their affiliated organizations, or those of the publisher, the editors and the reviewers. Any product that may be evaluated in this article, or claim that may be made by its manufacturer, is not guaranteed or endorsed by the publisher.

## Supplementary Material

Supplementary Figure 1 Multiple isoforms of NECAB2 in human, mouse, and zebrafish. **(A)** The protein diagrams of NECAB2 isoforms in human (*Homo sapiens*), mouse (*Mus musculus*), and zebrafish (*Danio rerio*) transcripts were shown, predicted from the Ensemble database. Scale bar = 50 amino acids (aa). **(B)** Transcript-specific primers were generated to confirm the existence of the two predicted transcripts of *necab2* in the Ensemble database. cDNA is obtained from AB zebrafish larvae at 1 hpf, 10 hpf, 2 dpf, 3 dpf, 4 dpf, and 5 dpf, respectively. The housekeeping gene β*-actin* was used as an internal reference. **(C)** Western blot analysis of Necab2 in the *necab2*^+/+^ and *necab2*^–/^*^–^* larvae. Note the two isoforms of Necab2 in zebrafish (arrows). Necab2-001 is supposed to be 47.91 kDa. Necab2-201 is supposed to be 30.25kDa. The bands detected both in the *necab2*^+/+^ and *necab2*^–/^*^–^* larvae were likely to be unspecific binding. hpf, hour post-fertilization.

Supplementary Figure 2 Specificity of the RNA probes and antibodies used in this study. **(A)** Self-designed anti-sense and sense probe of *necab2* were used in whole-mount *in situ* hybridization from 24 to 120 hpf. The contrast of *in situ* hybridization signals between the anti-sense and sense-probe was evident, which confirmed the specificity of the RNA probe. Scale bar = 500 μm. **(B)** Custom-produced rabbit anti-Necab2 polyclonal antibody was performed in immunofluorescence in both the *necab2*^+/+^ and *necab2^–/–^* larvae at 120 hpf. The contrast of immunofluorescent signals between the WT and the *necab2* mutant was evident though part of the forebrain showed slight residual immunoreactivity. This supported the specificity of the antibody with little cross-reactivity to Necab1 and Necab3. Scale bar = 100 μm. The region in the dashed white box was shown at higher magnification below. Scale bar = 20 μm. **(C)** Immuno-depletion analysis of the polyclonal rat anti-GRM1a antibody in the *necab2*^+/+^ larvae at 120 hpf. The mixture of anti-Grm1a antibody and the Grm1a antigen (1:5) eliminated the immunofluorescent signaling in the anti-Grm1a antibody staining alone, suggesting that the immunoreactivity of anti-GRM1a antibody staining was specific. Scale bar = 100 μm. The region in the dashed white box was shown at higher magnification below. Scale bar = 20 μm. hpf, day post-fertilization; Tel, telencephalon; Ce, cerebellum; Ha, habenula; OT, optic tectum; HB, hindbrain.

Supplementary Figure 3 Tg(*Pnecab2:EGFP*) under the control of the upstream regulatory elements of *necab2* manifests good fidelity. **(A)** Comparison of transgenic EGFP expression with *in situ* hybridization in 120 hpf *necab2*^+/+^ larvae. Scale bar = 100, 200 μm, respectively. **(B)** Co-immunostaining of the anti-Necab2 and anti-EGFP antibody in Tg(*Pnecab2:EGFP*) from whole-mount scale to cell-resolution. Scale bar = 50 μm. **(C)** Imaging at cellular resolution detected the necab*2*-expressing neuron projection from habenula in the diencephalon to the inter-peduncular nucleus (IPN) and expression in the spinal cord and retinal ganglion (arrowheads). Scale bar = 10, 50 μm, respectively. hpf, day post-fertilization; Tel, telencephalon; Di, diencephalon; Ce, cerebellum; Re, retina; Ha, habenula; OT, optic tectum; MHB, midbrain-hindbrain boundary; HB, hindbrain; lens, crystalline lens.

Supplementary Figure 4 *necab2* expression is not detected in glycinergic, dopaminergic, or serotonergic neurons. **(A–A″′)** *necab2* was not detected in glycinergic neurons. High single-cell resolution images in the cerebellum of Tg(*Pnecab2:EGFP*) detected no co-localization between *necab2* with *glyt2*. Single section. Z-stack, Maximum intensity projection. Scale bar = 50 μm. **(B–B″,C–C″)** *necab2* was not detected in dopaminergic neurons. Co-immunofluorescent staining of anti-EGFP with anti-TH in Tg(*Pnecab2:EGFP*) showed no co-localization. Scale bar = 50 μm. **(D–D″)** *necab2* was not detected in serotonergic neurons. Co-immunofluorescent staining of anti-EGFP with anti-5-HT in Tg(*Pnecab2:EGFP*) showed no co-localization. Scale bar = 50 μm. hpf, day post-fertilization; Tel, telencephalon; Ce, cerebellum; Ha, habenula; OT, optic tectum; HB, hindbrain.

Supplementary Figure 5 Verification of the co-immunoprecipitation products used for mass spectrometry. **(A)** Coomassie blue staining analysis. The *necab2*^+/+^ and *necab2*^–/^*^–^* zebrafish were processed for immunoprecipitation using the polyclonal rabbit anti-Necab2 antibody and analyzed by Coomassie blue staining. Two biological replicates were performed for each group. **(B)** Western blot analysis. The *necab2*^+/+^ and *necab2*^–/^*^–^* zebrafish were processed for immunoprecipitation by rabbit anti-Necab2 antibody and the crude extracts (Input), as well as immunoprecipitations (IP), were analyzed by the SDS-PAGE. Two biological replicates were performed for each group. Note that two NECAB2 isoforms (arrows) were identified by the antibody only in *necab2*^+/+^ but not the *necab2*^–/^*^–^* zebrafish.

Supplementary Figure 6 The expression of mGluR1 and the nature of neuronal subtypes expressing Necab2 in the larval zebrafish cerebellum. **(A–A″′)** Confocal live imaging of the *necab2*^+/+^ larvae fish at 5 dpf in Tg(*Pnecab2:EGFP*) and Tg(*gad1b:DsRed*) background in the cerebellar gad1b-enriched area. The Necab2-expressing neurons strongly overlapped with the gad1b-positive neurons (arrowheads). Scale bar = 100 μm **(A)**. The region in the dashed white box **(A)** was shown at higher magnification on the right **(A′–A″′)**. Scale bar = 10 μm. **(B–B″′)** Confocal live imaging of the *necab2*^+/+^ larvae fish at 5 dpf in Tg(*Pnecab2:EGFP*) and Tg(*vglut2a:DsRed*) background in the cerebellar gad1b-enriched area. The Necab2-expressing neurons overlapped with the vglut2a-positive neurons (arrowheads). Scale bar = 100 μm **(B)**. The region in the dashed white box **(B)** was shown at higher magnification on the right **(B′–B″′)**. Scale bar = 10 μm. **(C–C″′)** Confocal live imaging of the *necab2*^+/+^ larvae fish at 5 dpf in Tg(*Pnecab2:EGFP*) and Tg(*vglut2a:DsRed*) background in the cerebellar vglut2a-enriched area. The Necab2-expressing neurons overlapped with the vglut2a-positive neurons (arrowheads). Scale bar = 100 μm **(C)**. The region in the dashed white box **(C)** was shown at higher magnification on the right **(C′–C″′)**. Scale bar = 10 μm. **(D,E)** Immunostaining of anti-Grm1a in the *necab2*^+/+^ and *necab2*^–/^*^–^* larval cerebellum showed no difference in the mGluR1 expression. Scale bar = 20 μm. hpf, day post fertilization; Tel, telencephalon; Ce, cerebellum; ce-gad1b, cerebellar gad1b enriched area; ce-vglut2a, cerebellar vglut2a enriched area; Ha, habenula; OT, optic tectum; HB, hindbrain.

Supplementary Figure 7 The quantification of the plasma/cytosol intensity in the *in vivo* PIP2 imaging by ImageJ. **(A)** The live fluorescence imaging of Tg(*hsp70:plc-ph-mCherry*) with the confocal microscope. Scale bar = 10 μm. **(B)** The outline of the selected cell was drawn manually. The mean fluorescent intensity was calculated by ImageJ, which is the mean fluorescent intensity of the whole cell (Area = 0.546, Mean value = 44.025). Scale bar = 2 μm. **(C)** The cytosol fluorescent intensity was calculated after the outline of the cytoplasm was sketched (Area = 0.355, Mean value = 33.500). The plasma fluorescent intensity was obtained by subtracting cytosol intensity from whole cell intensity: The plasma fluorescent intensity = (mean fluorescent intensity of whole cell * area of whole cell - mean fluorescent intensity of cytosol * area of cytosol)/(area of whole cell - area of cytosol). The plasma/cytosol intensity = mean fluorescent intensity of plasma/mean fluorescent intensity of cytosol. Scale bar = 2 μm.
