# Supplementary material for: Autism-Risk Gene necab2 Regulates Psychomotor and Social Behavior as a Neuronal Modulator of mGluR1 Signaling
Source: Front Mol Neurosci. 2022 Jul 13;15:901682. doi: 10.3389/fnmol.2022.901682 (PMC9326220; doi:10.3389/fnmol.2022.901682)
Supplement: Supplementary file 1 [file Table_1.DOCX]

Table S1. Summary of clinical relevance between *NECAB2* and autism.

| **Proband ID** | **11327.p1** | **HI2486** | **11327** |
| --- | --- | --- | --- |
| Publication | Sanders, S.J. et al. (2011) | Itsara, A. et al. (2010) | Sakai, Y. et al. (2011) |
| Location | Chr16:79738681- 84748965 | Chr16:82557318- 82683859 | chr16:79754696-84728270 |
| Mutation | CNV-deletion | CNV-deletion | CNV-deletion |
| Phenotype | Autism | Autism | Autism Spectrum |
| Sex | Male | Not stated | Not stated |
| Age | 11 | Not stated | Not stated |
| Inheritance | Inherited, maternal | Not stated | De novo |
| RefSeq Genes | *ADAD2, ATP2C2, BCMO1, C16orf74, CDH13, CMIP, COTL1, COX4I1, COX4NB, CRISPLD2, FAM92B, GAN, GINS2, HSBP1, HSD17B2, HSDL1, IRF8, KCNG4, KIAA0182, KIAA0513, KIAA1609, KLHL36, LRRC50, MBTPS1, MLYCD, MPHOSPH6,* ***NECAB2,*** *OSGIN1, PKD1L2, PLCG2, SDR42E1, SLC38A8, TAF1C, USP10, WFDC1, ZDHHC7* | *MBTPS1,* ***NECAB2****, OSGIN1, SLC38A8* | *DYNLRB2, CDYL2, CMC2, CENPN, ATMIN, C16orf46, GCSH, PKD1L2, BCO1, GAN, CMIP, PLCG2, SDR42E1, HSD17B2, MPHOSPH6, CDH13, HSBP1, MLYCD, OSGIN1,* ***NECAB2****, SLC38A8, MBTPS1, HSDL1, DNAAF1, TAF1C, ADAD2, KCNG4, WFDC1, ATP2C2, MEAK7, COTL1, KLHL36, USP10* |
