# Supplementary figures and images for: Autism-Risk Gene necab2 Regulates Psychomotor and Social Behavior as a Neuronal Modulator of mGluR1 Signaling
Source: Front Mol Neurosci. 2022 Jul 13;15:901682. doi: 10.3389/fnmol.2022.901682 (PMC9326220; doi:10.3389/fnmol.2022.901682)

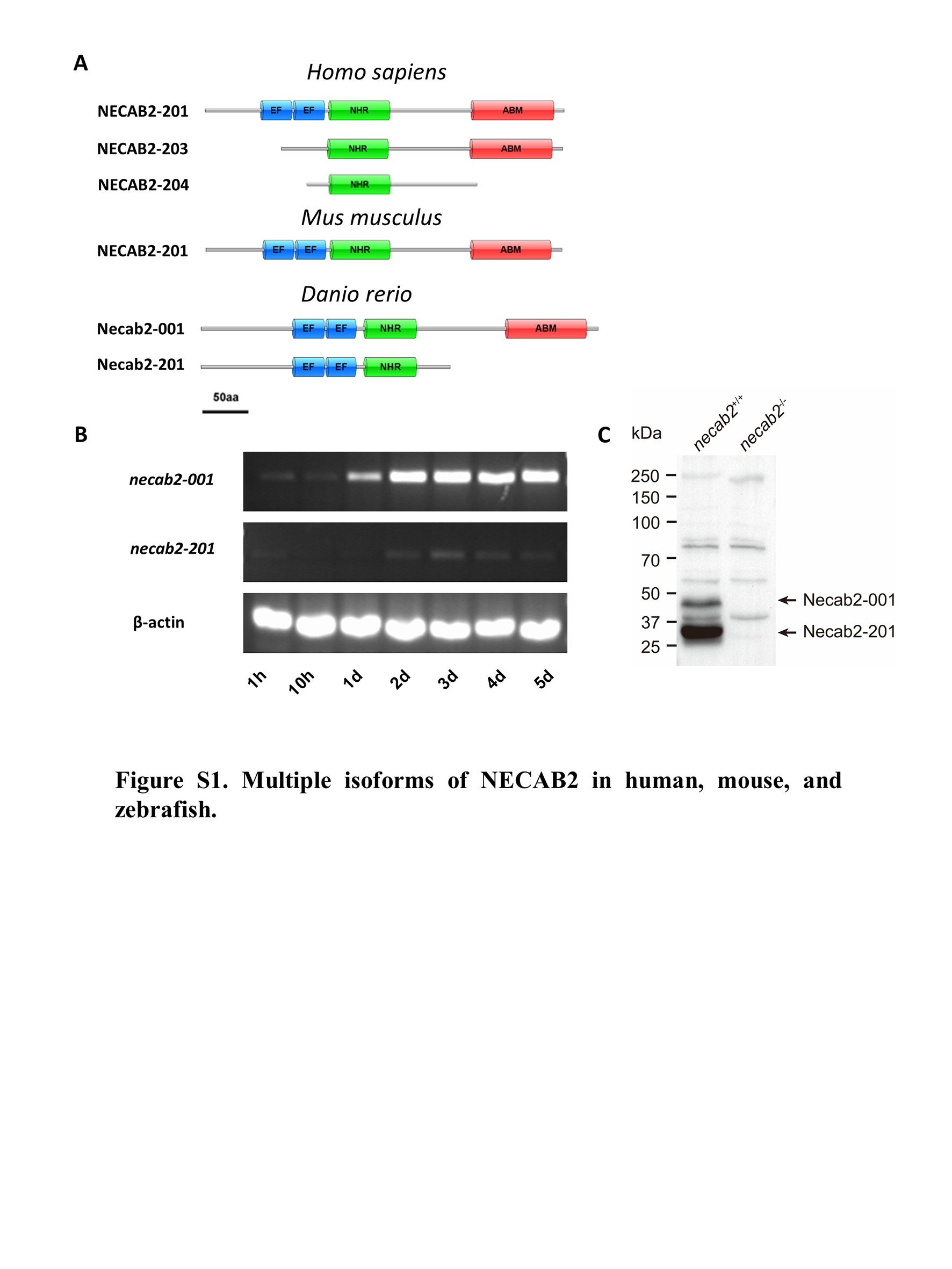

Supplement: Supplementary Figure 1 — Multiple isoforms of NECAB2 in human, mouse, and zebrafish. (A) The protein diagrams of NECAB2 isoforms in human (Homo sapiens), mouse (Mus musculus), and zebrafish (Danio rerio) transcripts were shown, predicted from the Ensemble database. Scale bar = 50 amino acids (aa). (B) Transcript-specific primers were generated to confirm the existence of the two predicted transcripts of necab2 in the Ensemble database. cDNA is obtained from AB zebrafish larvae at 1 hpf, 10 hpf, 2 dpf, 3 dpf, 4 dpf, and 5 dpf, respectively. The housekeeping gene β-actin was used as an internal reference. (C) Western blot analysis of Necab2 in the necab2+/+ and necab2–/– larvae. Note the two isoforms of Necab2 in zebrafish (arrows). Necab2-001 is supposed to be 47.91 kDa. Necab2-201 is supposed to be 30.25kDa. The bands detected both in the necab2+/+ and necab2–/– larvae were likely to be unspecific binding. hpf, hour post-fertilization. [file Image_1.TIF]

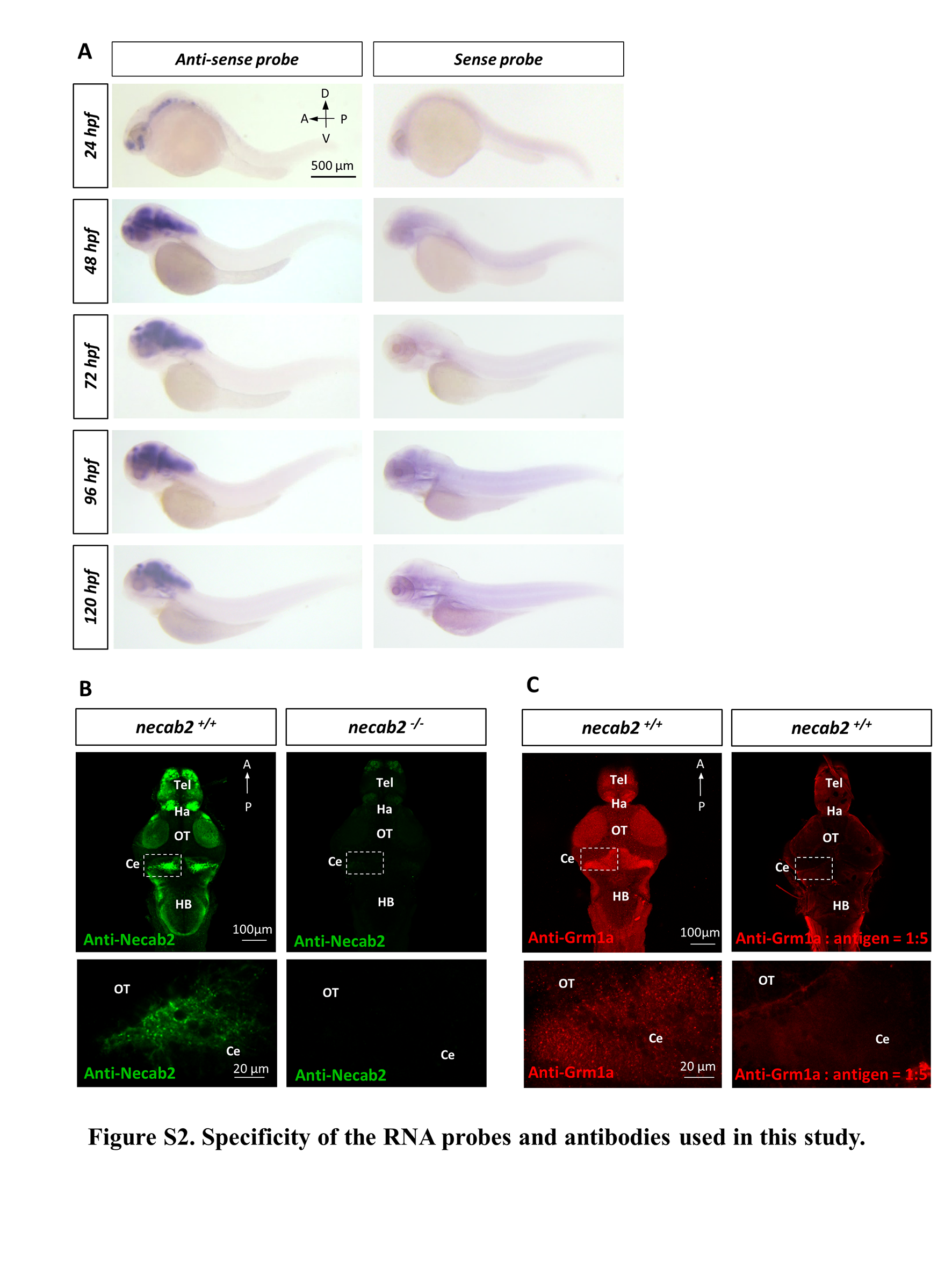

Supplement: Supplementary Figure 2 — Specificity of the RNA probes and antibodies used in this study. (A) Self-designed anti-sense and sense probe of necab2 were used in whole-mount in situ hybridization from 24 to 120 hpf. The contrast of in situ hybridization signals between the anti-sense and sense-probe was evident, which confirmed the specificity of the RNA probe. Scale bar = 500 μm. (B) Custom-produced rabbit anti-Necab2 polyclonal antibody was performed in immunofluorescence in both the necab2+/+ and necab2–/– larvae at 120 hpf. The contrast of immunofluorescent signals between the WT and the necab2 mutant was evident though part of the forebrain showed slight residual immunoreactivity. This supported the specificity of the antibody with little cross-reactivity to Necab1 and Necab3. Scale bar = 100 μm. The region in the dashed white box was shown at higher magnification below. Scale bar = 20 μm. (C) Immuno-depletion analysis of the polyclonal rat anti-GRM1a antibody in the necab2+/+ larvae at 120 hpf. The mixture of anti-Grm1a antibody and the Grm1a antigen (1:5) eliminated the immunofluorescent signaling in the anti-Grm1a antibody staining alone, suggesting that the immunoreactivity of anti-GRM1a antibody staining was specific. Scale bar = 100 μm. The region in the dashed white box was shown at higher magnification below. Scale bar = 20 μm. hpf, day post-fertilization; Tel, telencephalon; Ce, cerebellum; Ha, habenula; OT, optic tectum; HB, hindbrain. [file Image_2.TIF]

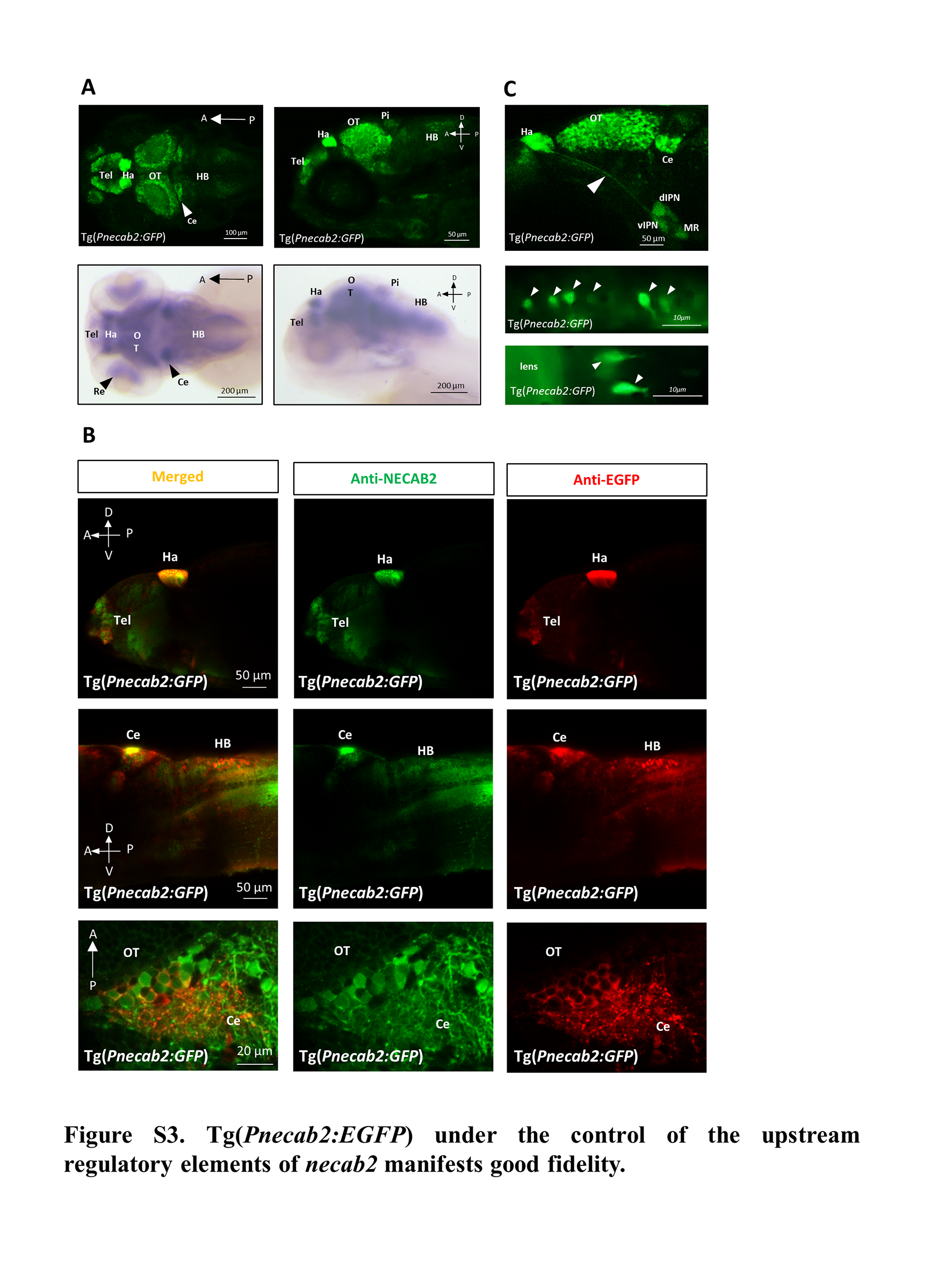

Supplement: Supplementary Figure 3 — Tg(Pnecab2:EGFP) under the control of the upstream regulatory elements of necab2 manifests good fidelity. (A) Comparison of transgenic EGFP expression with in situ hybridization in 120 hpf necab2+/+ larvae. Scale bar = 100, 200 μm, respectively. (B) Co-immunostaining of the anti-Necab2 and anti-EGFP antibody in Tg(Pnecab2:EGFP) from whole-mount scale to cell-resolution. Scale bar = 50 μm. (C) Imaging at cellular resolution detected the necab2-expressing neuron projection from habenula in the diencephalon to the inter-peduncular nucleus (IPN) and expression in the spinal cord and retinal ganglion (arrowheads). Scale bar = 10, 50 μm, respectively. hpf, day post-fertilization; Tel, telencephalon; Di, diencephalon; Ce, cerebellum; Re, retina; Ha, habenula; OT, optic tectum; MHB, midbrain-hindbrain boundary; HB, hindbrain; lens, crystalline lens. [file Image_3.TIF]

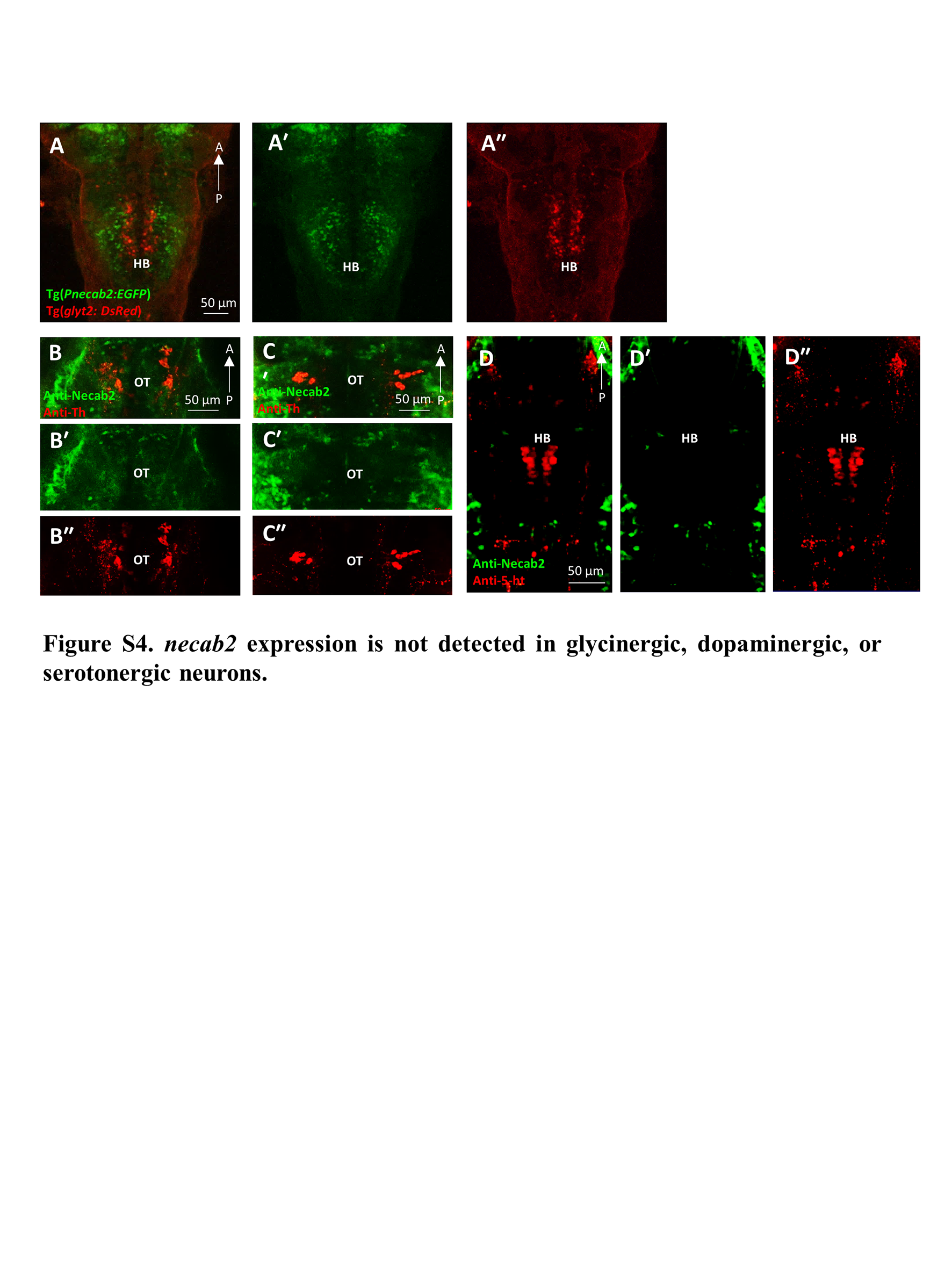

Supplement: Supplementary Figure 4 — necab2 expression is not detected in glycinergic, dopaminergic, or serotonergic neurons. (A–A″′) necab2 was not detected in glycinergic neurons. High single-cell resolution images in the cerebellum of Tg(Pnecab2:EGFP) detected no co-localization between necab2 with glyt2. Single section. Z-stack, Maximum intensity projection. Scale bar = 50 μm. (B–B″,C–C″) necab2 was not detected in dopaminergic neurons. Co-immunofluorescent staining of anti-EGFP with anti-TH in Tg(Pnecab2:EGFP) showed no co-localization. Scale bar = 50 μm. (D–D″) necab2 was not detected in serotonergic neurons. Co-immunofluorescent staining of anti-EGFP with anti-5-HT in Tg(Pnecab2:EGFP) showed no co-localization. Scale bar = 50 μm. hpf, day post-fertilization; Tel, telencephalon; Ce, cerebellum; Ha, habenula; OT, optic tectum; HB, hindbrain. [file Image_4.TIF]

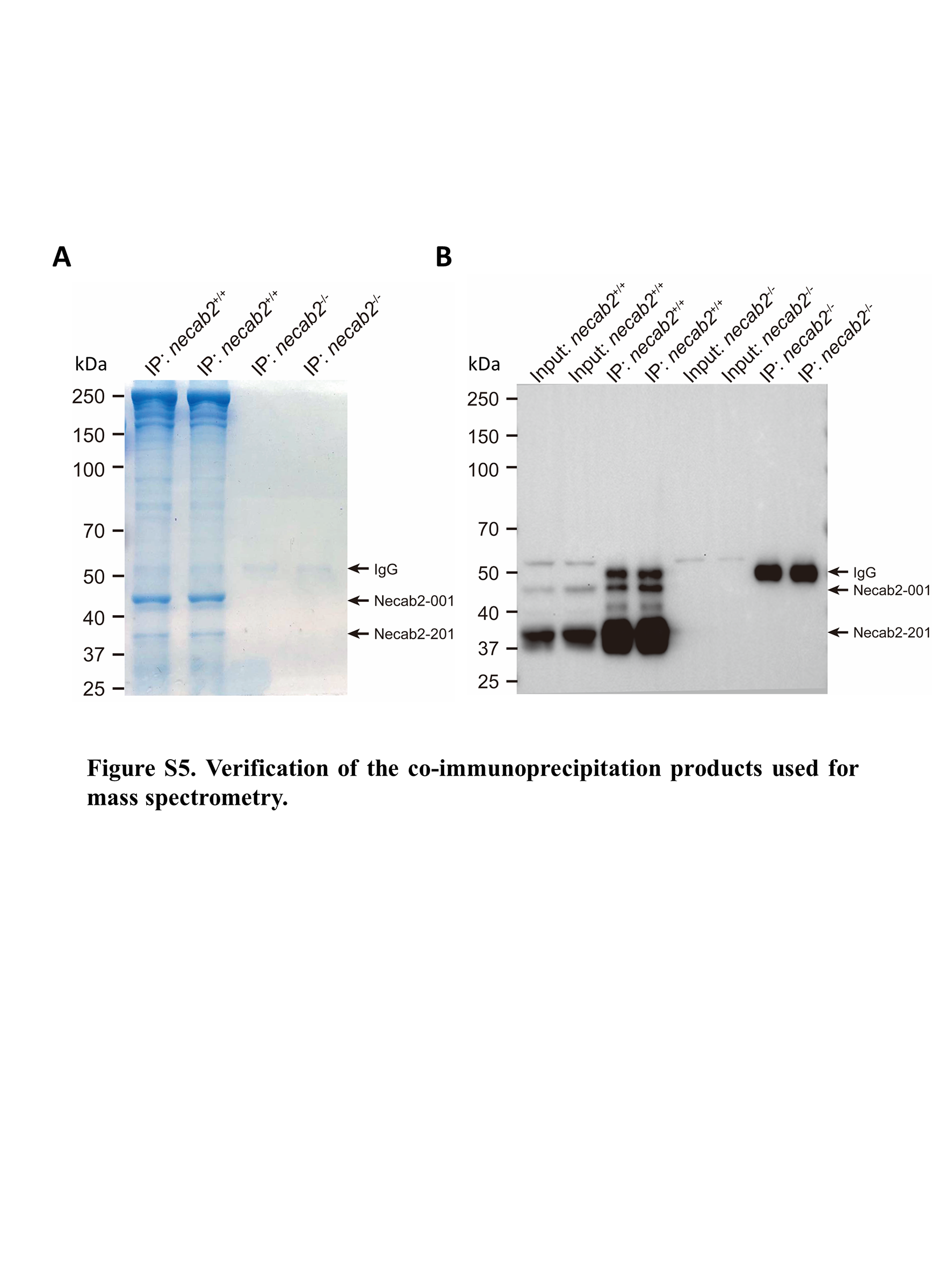

Supplement: Supplementary Figure 5 — Verification of the co-immunoprecipitation products used for mass spectrometry. (A) Coomassie blue staining analysis. The necab2+/+ and necab2–/– zebrafish were processed for immunoprecipitation using the polyclonal rabbit anti-Necab2 antibody and analyzed by Coomassie blue staining. Two biological replicates were performed for each group. (B) Western blot analysis. The necab2+/+ and necab2–/– zebrafish were processed for immunoprecipitation by rabbit anti-Necab2 antibody and the crude extracts (Input), as well as immunoprecipitations (IP), were analyzed by the SDS-PAGE. Two biological replicates were performed for each group. Note that two NECAB2 isoforms (arrows) were identified by the antibody only in necab2+/+ but not the necab2–/– zebrafish. [file Image_5.TIF]

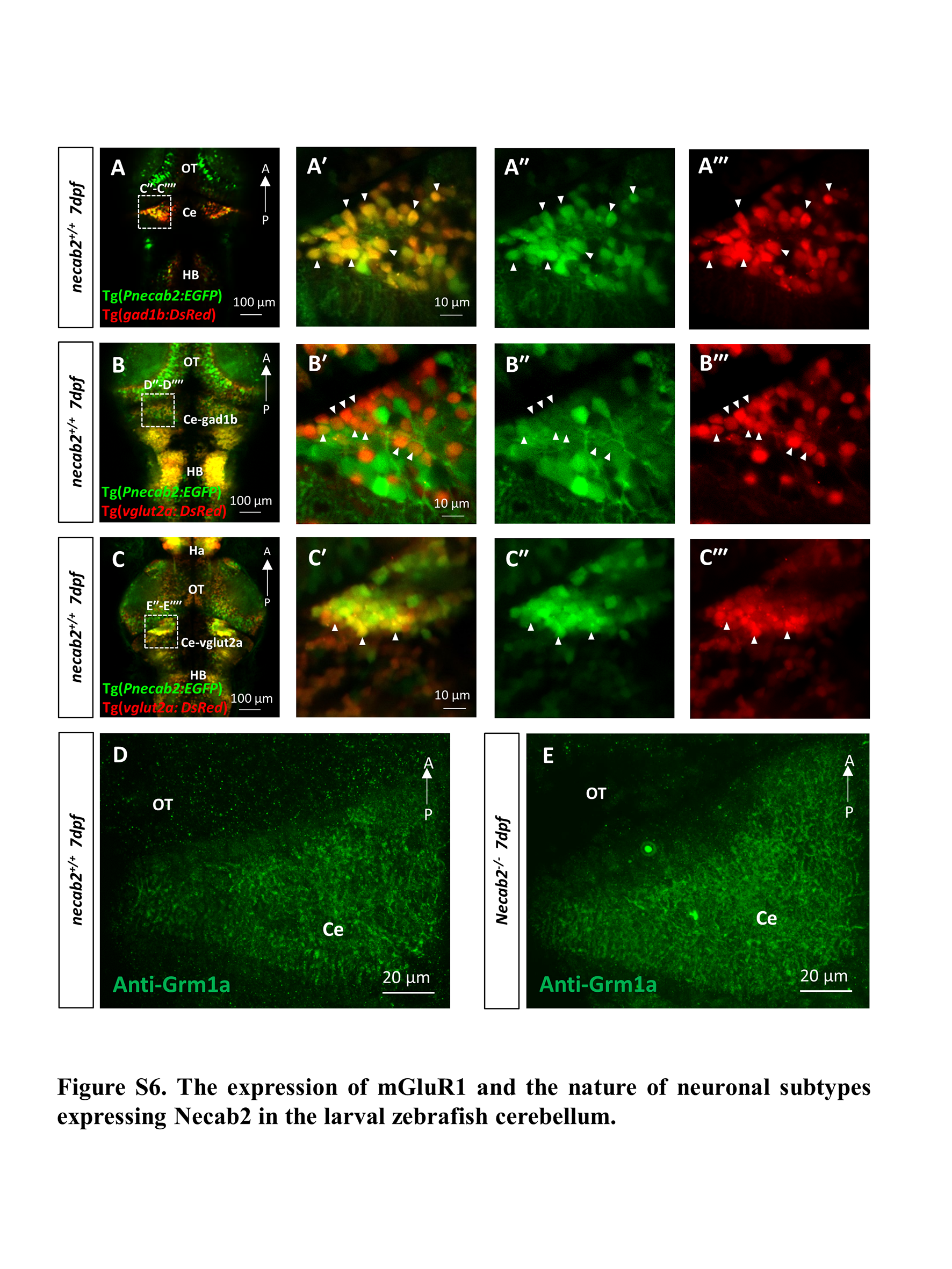

Supplement: Supplementary Figure 6 — The expression of mGluR1 and the nature of neuronal subtypes expressing Necab2 in the larval zebrafish cerebellum. (A–A″′) Confocal live imaging of the necab2+/+ larvae fish at 5 dpf in Tg(Pnecab2:EGFP) and Tg(gad1b:DsRed) background in the cerebellar gad1b-enriched area. The Necab2-expressing neurons strongly overlapped with the gad1b-positive neurons (arrowheads). Scale bar = 100 μm (A). The region in the dashed white box (A) was shown at higher magnification on the right (A′–A″′). Scale bar = 10 μm. (B–B″′) Confocal live imaging of the necab2+/+ larvae fish at 5 dpf in Tg(Pnecab2:EGFP) and Tg(vglut2a:DsRed) background in the cerebellar gad1b-enriched area. The Necab2-expressing neurons overlapped with the vglut2a-positive neurons (arrowheads). Scale bar = 100 μm (B). The region in the dashed white box (B) was shown at higher magnification on the right (B′–B″′). Scale bar = 10 μm. (C–C″′) Confocal live imaging of the necab2+/+ larvae fish at 5 dpf in Tg(Pnecab2:EGFP) and Tg(vglut2a:DsRed) background in the cerebellar vglut2a-enriched area. The Necab2-expressing neurons overlapped with the vglut2a-positive neurons (arrowheads). Scale bar = 100 μm (C). The region in the dashed white box (C) was shown at higher magnification on the right (C′–C″′). Scale bar = 10 μm. (D,E) Immunostaining of anti-Grm1a in the necab2+/+ and necab2–/– larval cerebellum showed no difference in the mGluR1 expression. Scale bar = 20 μm. hpf, day post fertilization; Tel, telencephalon; Ce, cerebellum; ce-gad1b, cerebellar gad1b enriched area; ce-vglut2a, cerebellar vglut2a enriched area; Ha, habenula; OT, optic tectum; HB, hindbrain. [file Image_6.TIF]

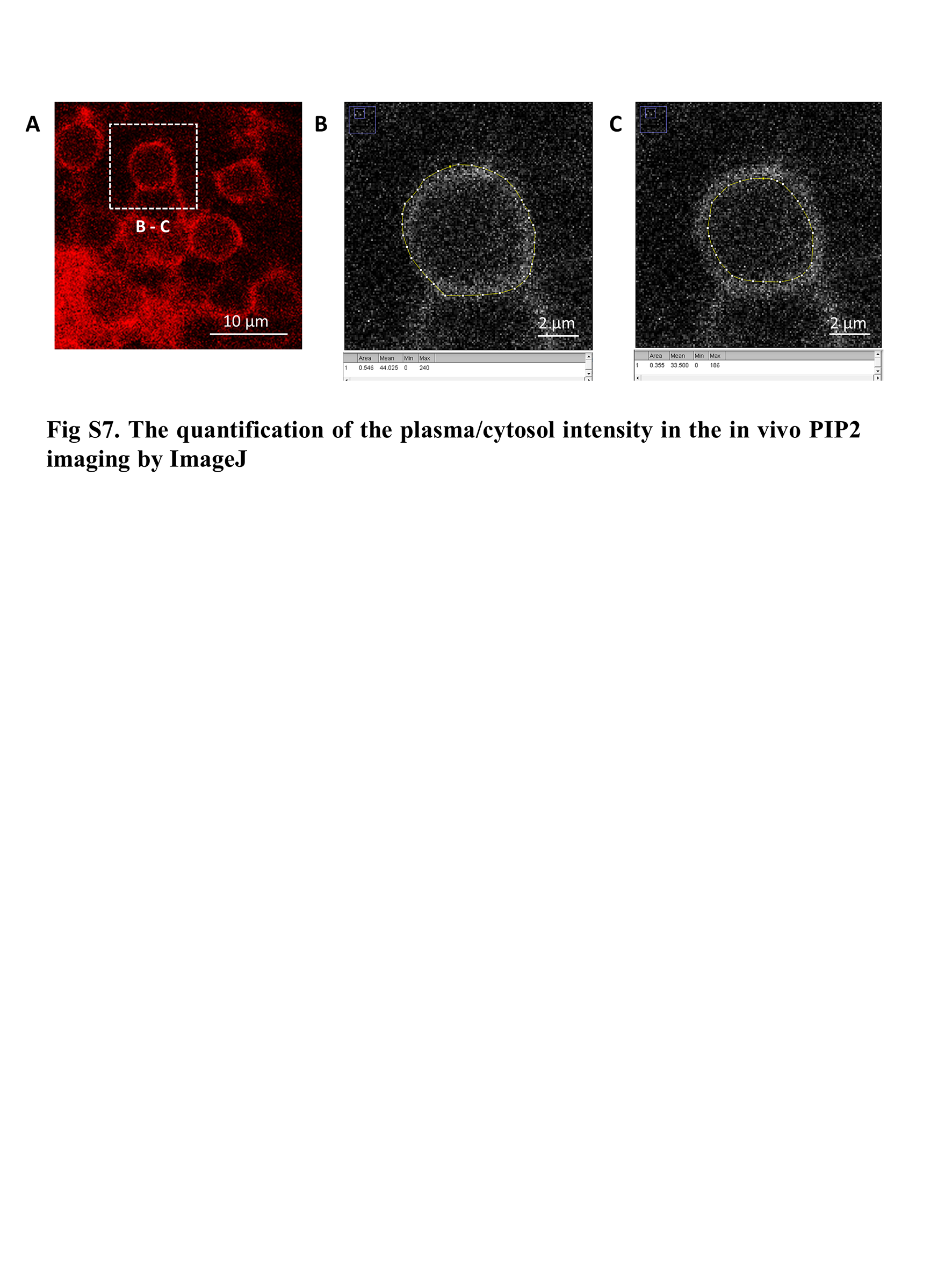

Supplement: Supplementary Figure 7 — The quantification of the plasma/cytosol intensity in the in vivo PIP2 imaging by ImageJ. (A) The live fluorescence imaging of Tg(hsp70:plc-ph-mCherry) with the confocal microscope. Scale bar = 10 μm. (B) The outline of the selected cell was drawn manually. The mean fluorescent intensity was calculated by ImageJ, which is the mean fluorescent intensity of the whole cell (Area = 0.546, Mean value = 44.025). Scale bar = 2 μm. (C) The cytosol fluorescent intensity was calculated after the outline of the cytoplasm was sketched (Area = 0.355, Mean value = 33.500). The plasma fluorescent intensity was obtained by subtracting cytosol intensity from whole cell intensity: The plasma fluorescent intensity = (mean fluorescent intensity of whole cell * area of whole cell - mean fluorescent intensity of cytosol * area of cytosol)/(area of whole cell - area of cytosol). The plasma/cytosol intensity = mean fluorescent intensity of plasma/mean fluorescent intensity of cytosol. Scale bar = 2 μm. [file Image_7.tif]
